# Phosphate Framework Electrode Materials for Sodium Ion Batteries

**DOI:** 10.1002/advs.201600392

**Published:** 2017-01-18

**Authors:** Yongjin Fang, Jiexin Zhang, Lifen Xiao, Xinping Ai, Yuliang Cao, Hanxi Yang

**Affiliations:** ^1^ College of Chemistry and Molecular SciencesHubei Key Laboratory of Electrochemical Power SourcesWuhan UniversityWuhan430072P.R. China; ^2^ College of ChemistryCentral China Normal UniversityWuhan430079P.R. China

**Keywords:** electrode materials, energy storage, Na‐ion batteries, phosphate, polyanions

## Abstract

Sodium ion batteries (SIBs) have been considered as a promising alternative for the next generation of electric storage systems due to their similar electrochemistry to Li‐ion batteries and the low cost of sodium resources. Exploring appropriate electrode materials with decent electrochemical performance is the key issue for development of sodium ion batteries. Due to the high structural stability, facile reaction mechanism and rich structural diversity, phosphate framework materials have attracted increasing attention as promising electrode materials for sodium ion batteries. Herein, we review the latest advances and progresses in the exploration of phosphate framework materials especially related to single‐phosphates, pyrophosphates and mixed‐phosphates. We provide the detailed and comprehensive understanding of structure–composition–performance relationship of materials and try to show the advantages and disadvantages of the materials for use in SIBs. In addition, some new perspectives about phosphate framework materials for SIBs are also discussed. Phosphate framework materials will be a competitive and attractive choice for use as electrodes in the next‐generation of energy storage devices.

## 1 Introduction

**Figure 1:**
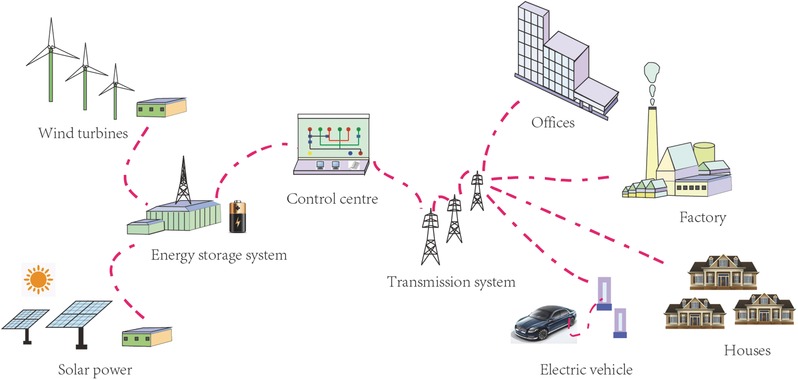
Schematic illustration of the “energy internet”.

Rapid growth of renewable electricity in global energy markets has continuously propelled the development of effective and affordable energy storage technologies for constructing a future energy internet (**Figure** **1**).^1^ Though battery technologies have been developed over a hundred of years, few of them can meet the needs for ever‐increasing electric energy storage applications. For example, Lithium ion batteries (LIBs) are now considered as a promising candidate for a number of electric storage applications, there exists a great concern about the widespread availability and rising price of lithium resources.^2^ Therefore, room temperature Na‐ion batteries (SIBs) appear to be a good choice as a viable technology for large scale electric storage applications due to their low cost, widespread abundance of sodium resources, cheap Al anode collector used and chemical similarity with Li‐ion batteries.

In the past few years, tremendous efforts have been made to exploring suitable Na‐host materials with high reversible capacity, rapid Na ion insertion/extraction and cycling stability.^3^, ^4^, ^5^ A large variety of compounds, such as transition metal oxides,^6^, ^7^, ^8^, ^9^ phosphates,^10^, ^11^, ^12^ ferrocyanide,^13^, ^14^, ^15^ hard carbon,^16^, ^17^, ^18^, ^19^ metal alloys,^20^, ^21^, ^22^ and organic materials,^23^, ^24^, ^25^ have demonstrated considerable Na‐storage capacities for SIBs. However, most of them suffer from structural instability during Na‐insertion/extraction reactions. For example, the Na ion insertion/extraction in the layered metal oxides often results in very complicated multiphase transitions, leading to rapid structural degradation of the hosts during cycling.^7^ In addition, they are very sensitive to atmosphere and water, making the large scale application of these materials a severe problem.^5^ For the metal alloy anodes, they possess high reversible capacity and huge volumetric expansion (for example, a 420% volumetric expansion for Na_3.75_Sn to Sn) during Na insertion, resulting in exfoliation and inactivation of the active materials.^26^

From the viewpoint of structural stability, open framework favors to accommodate the large‐sized Na ions. For example, the Prussian blue analogues have a cubic framework capable of reversible extraction of two Na per formula unit at high rates. However, these compounds from conventional synthesis always contain a large amount of lattice defects and coordinated water, which cause a huge loss of the active sites for Na ion storage.^14^, ^27^ Additionally, the thermal unstable structure also arise concerns about the safety issues of the materials for application. In this respect, transition metal compounds containing polyanions such as PO_4_^3−^ are intensively investigated because the strong P‐O covalent bonds can stabilize the lattice oxygen even at highly charged state. And the phosphate framework materials show very low thermal expansion (the coefficient of thermal expansion is around 10^–6^ °C^–1^),^28^ indicating high structural stability at high temperature.

Phosphate framework materials have attracted increasing attention due to their excellent electrochemical performance and versatile structure,^29^ and are considered as promising Na‐storage electrodes due to the following considerations: (i) phosphate frameworks have high structural stability due to the very stable P‐O frameworks, thus ensuring long‐term cycling and safety of SIBs; the thermal properties of phosphate materials are linked directly to the stability of the phosphate‐metal bonds, which greatly reduce the likelihood of oxygen liberation from the structure; (ii) the 3D framework possesses many roomy interstices, thus leading to lower volumetric expansion and less phase transition during Na ion insertion/extraction, which benefits the structural stability; (iii) phosphate or other substituent groups exhibit inductive effect on the redox couple, thus to give rise to higher redox potential values vs Na/Na^+^. However, the big size and intrinsic isolating nature of the PO_4_^3–^ groups lead to a moderate capacity and low electron conductivity. In this context, constructing elaborate structure with highly conductive matrix is an effective approach to improve high performance of the phosphate materials.

Phosphate framework materials are full of variety, exhibiting versatile and adjustable structure and electrochemical performance, such as phosphates, pyrophosphates, mixed‐anions, and diverse optional redox centers (Fe, V, Mn, Ni, Co, et al.). From the viewpoint of electrochemistry, most of the reactions are attributed to phase transition mechanism, and some belong to solid solution, surface or interface charging mechanisms.

In this review, recent research progress on the use of phosphate framework materials for SIBs is summarized, concentrating especially on single‐phosphates, pyrophosphates and mixed‐phosphates. We provide a detailed and comprehensive understanding of structure–composition–performance relationship of the materials and try to reveal the advantages and disadvantages of the materials for SIBs. In addition, some new perspectives about phosphate framework materials for SIBs are also discussed.

## 2 Single‐Phosphate Materials for Na Storage

Single‐phosphate materials are the first to be investigated as electrode materials for SIBs. A large variety of phases used in Li ion intercalation chemistry have been investigated as drop‐in replacements for Na ion. Among the phosphates materials, olivine structure, NASICON‐type materials and other kind of materials have attracted much more attention due to their decent electrochemical performance.

### 2.1 Olivine and Maricite Structure

#### 2.1.1 Olivine Structure

**Figure 2:**
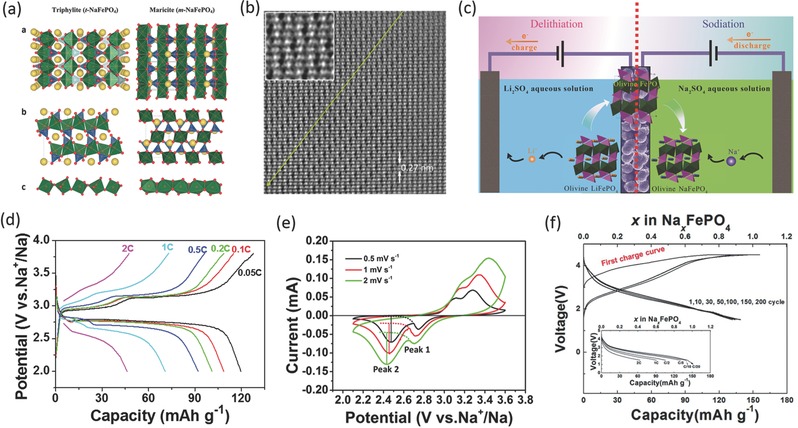
(a) Schematic presentation of orthorhombic structured triphylite NaFePO_4_ (left) and maricite NaFePO_4_ (right) polymorphs; (b) STEM image of Na_≈2/3_FePO_4_. The arrow line highlights the orientation of the Na−vacancies (black dots); (c) Synthetic scheme of the aqueous electrochemical displacement process from olivine LiFePO_4_ to isostructural NaFePO_4_; (d) The voltage‐capacity profiles of the NaFePO_4_/C electrode at different charge/discharge rates from 0.05 to 2 C; (e) Cyclic voltammograms of NaFePO_4_/C electrode in 1 mol L^–1^ NaPF_6_/EC: DEC (1:1 in vol.) solution at various scan rates; (f) Galvanostatic curves of maricite NaFePO_4_ over 200 cycles at C/20 in a Na cell, inset: discharge curves of maricite NaFePO_4_ as a function of the C rate from C/20 to 3 C (charging under CCCV mode (C/20 rate and 5 hour holding at 4.5 V)), during the first charge of CV, 20 mAh g^–1^ of capacity was recovered. (a) Reproduced with permission.^55^ Copyright 2013, American Chemical Society. (b) Reproduced with permission.^38^ Copyright 2014, American Chemical Society. (c, d, e) Reproduced with permission.^44^ Copyright 2015, American Chemical Society. (f) Reproduced with permission.^57^ Copyright 2015, Royal Society of Chemistry.

As LiFePO_4_ is successfully commercialized as a cathode material for lithium ion battery applications, its sodium analogue, olivine NaFePO_4_, has attracted interest as a Na ions host material due to its high theoretical specific capacity (154 mAh g^–1^) and decent voltage (≈2.8 V). However, direct high‐temperature synthesis cannot produce pure olivine‐phased NaFePO_4_ but usually lead to a thermodynamically favored maricite phase, which has poor electrochemical activity because its one‐dimensional, edge‐sharing FeO_6_ octahedrons form frustrated pathways for Na‐ion migration (**Figure** **2**a).^30^, ^31^, ^32^

The conversion from a maricite phase to an olivine phase by cation exchange seems to be a feasible way to obtain pure olivine NaFePO_4_. Le Poul et al. first studied the Na ion insertion behavior of olivine FePO_4_ and found that 0.65 sodium ion can be inserted to form Na_0.65_FePO_4_ at 0.1 C rate.^33^ After that, tremendous efforts have been focused on the synthesis and characterization of olivine NaFePO_4_ material for SIBs. Moreau et al. synthesized the Na_x_FePO_4_ phases (x = 0.7 and 1) via electrochemical method and provided a detailed structural analysis of olivine NaFePO_4_,^31^ By examining Na intercalation behaviors in the olivine FePO_4_, they unraveled a stable immediate Na_2/3_FePO_4_ phase with a superstructure (Figure 2b).^34^, ^35^ The olivine NaFePO_4_ electrode exhibits two charge plateaus separated by a voltage drop corresponding to the intermediate Na_2/3_FePO_4_ phase, and one discharge plateau. Oh et al. have also studied the electrochemical performance of NaFePO_4_ obtained by an electrochemical exchange from LiFePO_4_, and obtained a stable capacity of 125 mAh g^–1^ with a cycle life of 50 cycles.^36^ This work demonstrates the possibility to achieve good Na storage performance for olivine NaFePO_4_ material.

Casas‐Cabanas's group has done exhaustive investigation on the Na insertion/deinsertion behaviors of olivine NaFePO_4_. They found that the intermediate Na_2/3_FePO_4_ phase forms in both charge and discharge process and characterized the intermediate phase with Na/vacancies ordering.^37^, ^38^ The Na insertion and deinsertion occur through different mechanisms due to the huge volumetric mismatch between FePO_4_ and NaFePO_4_ (17.58% difference in unit volume).^39^ A Na_5/6_FePO_4_ intermediate phase was also revealed by DFT calculations and high resolution synchrotron X‐ray diffraction.^40^ Yamada's group determined the composition‐temperature phase diagram of the FePO_4_/NaFePO_4_ system and found that Na_x_FePO_4_ (0 < x < 2/3) appeared in a two‐phase region, while Na_x_FePO_4_ (2/3 < x < 1) is in solid‐solution region.^41^ Polaron mobility and disordering of the sodium sublattice and variation of local magnetic environments of Na_x_FePO_4_ have also been settled.^42^, ^43^

High‐performance olivine NaFePO_4_ electrodes were also reported. Fang et al. synthesized olivine NaFePO_4_ microsphere through aqueous electrochemical displacement method from LiFePO_4_ and showed excellent cycling stability of the material with 90% capacity retention over 240 cycles. They reported for the first time a Na_2/3_FePO_4_ intermediate phase during discharge process through conventional electrochemical techniques (Figure 2c–e).^44^ Tang et al. promoted the research by similar synthesis route and the staged evolution of phases during sodiation/desodiation were also studied.^45^ Ali et al. have modified the NaFePO_4_ with polythiophene and demonstrated that the discharge capacity and cycle life of NaFePO_4_ electrode can be greatly improved by polythiophene coating.^46^ Ionic liquid electrolytes with various sodium solutes were tested for olivine NaFePO_4_ by Chang's group. At elevated temperature (50 and 75 °C), the ionic liquid electrolytes assist NaFePO_4_ in delivering higher capacity with stable cycle life.^47^, ^48^

Structural and electrochemical features between olivine NaFePO_4_ and LiFePO_4_ have also been well investigated. Zhu et al. compared the electrochemical performances of olivine NaFePO_4_ and olivine LiFePO_4_ and attributed the more sluggish Na storage behavior of NaFePO_4_ to the lower Na ion diffusion coefficient and higher charge transfer resistance in NaFePO_4_ electrodes.^49^ Whiteside et al. computed the surface structures and equilibrium morphology of olivine NaFePO_4_ and compared with those of LiFePO_4_. They found that NaFePO_4_ differs from LiFePO_4_ in the detail of its surface structures and their relative energies, such that the equilibrium morphology is thinner in the b‐axis direction, which is important for the rate performance.^50^ Density functional studies of Li and Na diffusion in LiFePO_4_ and NaFePO_4_ were also conducted by Nakayama and Major's groups,^51^, ^52^ and they found that electronic and Li or Na ionic migration in the bulk did not show large difference between LiFePO_4_ and NaFePO_4_.The changes of phase structure indicated a two‐phase reaction mechanism for the Li_1–x_FePO_4_ electrode and a solid‐solution reaction mechanism for the Na_1–x_FePO_4_ electrode.^51^, ^53^

#### 2.1.2 Maricite and Alluaudite Structure

As discussed above, it is well accepted that the maricit NaFePO_4_ is electrochemically inactive due to the reversed phosphate framework compared to olivine structure, where the M1 and M2 sites are occupied by Fe^2+^ and Na^+^, respectively, resulting in no free channels for Na ion diffusion in the closed framework (Figure 2a).^30^, ^31^, ^32^, ^54^ The magnetic structures of maricite and olivine NaFePO_4_ were well studied through experimental test and theoretical computation.^55^, ^56^ However, maricite NaFePO_4_ was recently proved to enable excellent Na storage performance and all Na ions can be extracted from the nano‐sized maricite NaFePO_4_ with simultaneous transformation of the maricite structure to amorphous FePO_4_.^57^ The maricite NaFePO_4_ electrode can deliver a capacity of 142 mAh g^–1^ at 1/20 C with sloping charge/discharge curves and stable cycle life over 200 cycles (Figure 2f).

Huang et al. have synthesized an alluaudite Na_0.67_FePO_4_/CNT (Na_2_Fe_3_(PO_4_)_3_/CNT) material, which delivered a discharge capacity of 143 mAh g^–1^ with stable cyclability. However, the first charge capacity is a quite low (≈40 mAh g^–1^), which restricts its battery application.^58^ Alluaudite NaMnFe_2_(PO_4_)_3_ has also been investigated but its electrochemical performance needs to be improved.^59^

### 2.2 NASICON‐type Na_x_M_2_(PO_4_)_3_ (M = V, Ti, 1 ≤ x ≤ 3)

**Figure 3:**
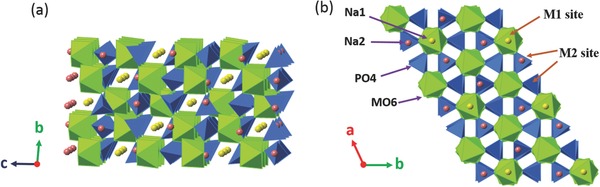
A view of the Na_x_M_2_(PO_4_)_3_ NASICON structure along the a (a) and c (b) axis, respectively.

To obtain electrodes with high‐performance and stable cyclability, unique crystal structure with roomy interstices and large tunnels, such as the 3D framework compounds that possess fast Na ions transport routes, are welcomed to accommodate these large sodium ions during a lattice perturbation‐free intercalation/deintercalation process.^60^ In this regard, NASICON (Na Super Ionic Conductor) type materials are well known for their high Na ionic conductivity and stable 3D framework and have been widely investigated as battery materials (electrodes, solid electrolytes, and membranes).^61^ The NASICON structure can be described as a covalent skeleton [M_2_P_3_O_12_]^–^ consisting of MO_6_ octahedra and PO_4_ tetrahedra, which form 3D interconnected tunnels and two types of interstitial positions (M1 and M2) where Na ions are distributed (**Figure** **3**).^62^, ^63^, ^64^ Generally, the compounds crystallize with a thermally stable rhombohedral structure, where the Na ions move from one site to another through bottlenecks.^63^ But members of A_3_M_2_(PO_4_)_3_ family (where A = Li, Na and M = Cr, Fe, Zr) crystallize in monoclinic modification and show reversible structural phase transitions at high temperatures.^65^, ^66^ As electrode materials, the NASICON Na_x_M_2_(PO_4_)_3_ (M = V, Fe, Ti, 0 ≤ x ≤ 3) have been widely studied, based on the consideration that the NASICON‐type lattices ensures long term cycle life as well as high rate capability.

#### 2.2.1 NASICON‐type Na_3_V_2_(PO_4_)_3_

Na_3_V_2_(PO_4_)_3_, first reported by Porter et al.,^67^ has a 3D framework of VO_6_ octahedra sharing all of its corners with PO_4_ tetrahedra and one Na^+^ ion occupies the M1 sites with sixfold coordination and the other two Na^+^ ions occupy the M2 sites forming eightfold coordination. Only Na ions residing at M2 sites can be extracted for electrochemical reaction due to the weak bonding to surrounding oxygen atoms (Figure 3), giving a theoretical capacity of 117 mAh g^–1^ and a flat plateau at around 3.3 V (vs Na/Na^+^).^68^ When discharge to a low potential (<1.5 V), an additional Na^+^ can be inserted at M2 sites, achieving a fully occupied Na_4_V_2_(PO_4_)_3_ state. Therefore, the Na_3_V_2_(PO_4_)_3_ can either be used as cathode or anode materials.^69^ The Na_3_V_2_(PO_4_)_3_ material can also maintain good thermal stability up to 450 °C even in the desodiated state.^69^ The crystal structure of the NASICON Na_3_V_2_(PO_4_)_3_ phase has been investigated as a function of temperature by Chotard's group, who demonstrated that the Na_3_V_2_(PO_4_)_3_ displayed four distinct crystal structures in the range of –30 °C to 225 °C, named the α‐, β‐, β′‐, and γ‐NVP.^70^

**Figure 4:**
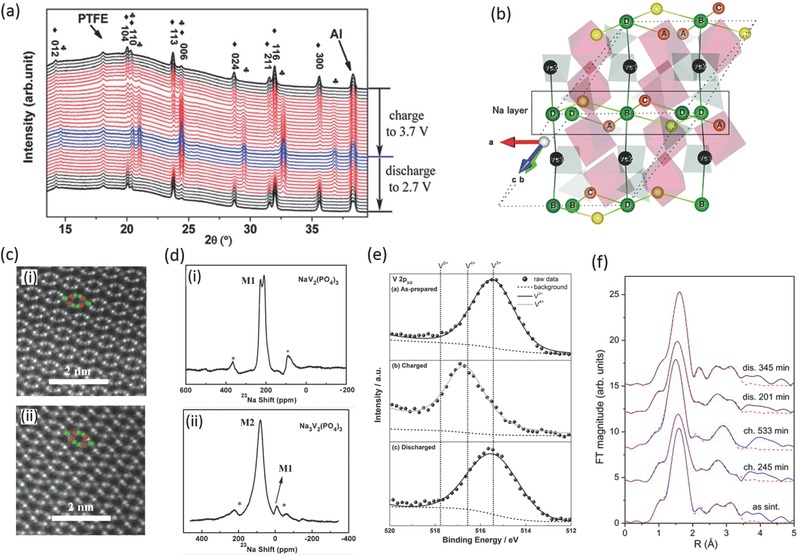
(a) In situ XRD patterns of the Na_3_V_2_(PO_4_)_3_/Na cell cycled between 3.7 and 2.7 V at a current rate of C/10, ♦ Na_3_V_2_(PO_4_)_3_, ♣ NaV_2_(PO_4_)_3_; (b) Schematic depiction of pathways 1, 2 and 3. The black‐green lines that connect Na at the Na1 site and the Na vacancy represent pathway 3; (c) STEM HAADF images of (i) Na_3_V_2_(PO_4_)_3_ and (ii) NaV_2_(PO_4_)_3_ along the $\left[1\bar{1}\bar{1}1\bar{1}\bar{1}\right]$ projection; (d) The ^23^Na MAS spectra for Na_3_V_2_(PO_4_)_3_ and NaV_2_(PO_4_)_3_ recorded at the spinning rate of 14 kHz at 298 K; (e) Ex‐situ XPS studies of NaV_2_(PO_4_)_3_/C electrodes; (f) Selected Fourier transform spectra of k^3^‐weighted V EXAFS spectra of the NaV_2_(PO_4_)_3_ sample during the first cycle of charge and discharge. Experiment–solid line; EXAFS model–dashed line. (a) Reproduced with permission.^74^ Copyright 2012, Wiley‐VCH. (b) Reproduced with permission.^77^ Copyright 2015, Royal Society of Chemistry. (c,d) Reproduced with permission.^75^ Copyright 2014, Wiley‐VCH. (e) Reproduced with permission.^60^ Copyright 2012, Wiley‐VCH. (f) Reproduced with permission.^79^ Copyright 2012, Elsevier.

Yamaki group first studied the Na storage performance of Na_3_V_2_(PO_4_)_3_ and obtained a reversible capacity of 140 mAh g^–1^ in the voltage range of 1.2–3.5 V.^71^ They constructed a symmetric cell using Na_3_V_2_(PO_4_)_3_ as both cathode and anode materials and ionic liquid as electrolyte, which delivered a reversible capacity of 64 mAh g^–1^.^72^ Due to the intrinsic low conductivity of the phosphate framework, the cycling stability and reversible capacity of the Na_3_V_2_(PO_4_)_3_ material appeared unsatisfactory. Hu's group realized the importance of improving conductivity of this material and used the carbon coating to obtain a Na_3_V_2_(PO_4_)_3_/C material.^73^ The Na_3_V_2_(PO_4_)_3_/C electrode delivered a reversible capacity of 93 mAh g^–1^ with improved cycling performance. They also optimized the carbon content and electrolyte system and significantly improved the Na storage performance, such as capacity, coulombic efficiency and cyclability (**Figure** **4**a).^74^ After that, they continued to investigate the Na^+^ ion positions at atomic resolution and the kinetics by using Rietveld refined‐XRD, ABF‐STEM (Figure 4c), and NMR (Figure 4d). The experiments exhibited that there were two kinds of Na sites in Na_3_V_2_(PO_4_)_3_ with different coordination environments, and only Na at the M2 sites can be extracted during electrochemical/chemical oxidation at room temperature, suggesting a M2‐M2 conduction pathway.^75^

The Na storage mechanism of Na_3_V_2_(PO_4_)_3_ has been well investigated by various spectroscopic, electrochemical and computing techniques. Song et al. used first principle calculations to explore the Na ion migration pathways and occupations. According to their study, two pathways along the x and y directions and one possible curved route for migration were favored with a 3D transport characteristics, and the ion occupation of 0.75 for all Na sites was suitable for the configuration of [Na_3_V_2_(PO_4_)_3_]_2_.^68^, ^76^ The crystal and electronic structures, electrochemical properties and diffusion mechanism of NASICON‐type Na_3_V_2_(PO_4_)_3_ have been investigated based on the hybrid density functional Heyd–Scuseria–Ernzerhof (HSE06) by Ohno's group. Three diffusion pathways, bound polaron behavior and activation barriers are revealed (Figure 4b).^77^ Yamada's group has measured the reaction entropy of the biphasic reaction of Na_1+2x_V_2_(PO_4_)_3_ using the potentiometric method and found that the reaction entropy is almost constant for 0.1 ≤ *x* ≤ 0.9.^78^ X‐ray absorption and electron paramagnetic resonance were also introduced to determine the local environment of the Na_3_V_2_(PO_4_)_3_ material at different charge states (Figure 4f).^79^, ^80^

**Figure 5:**
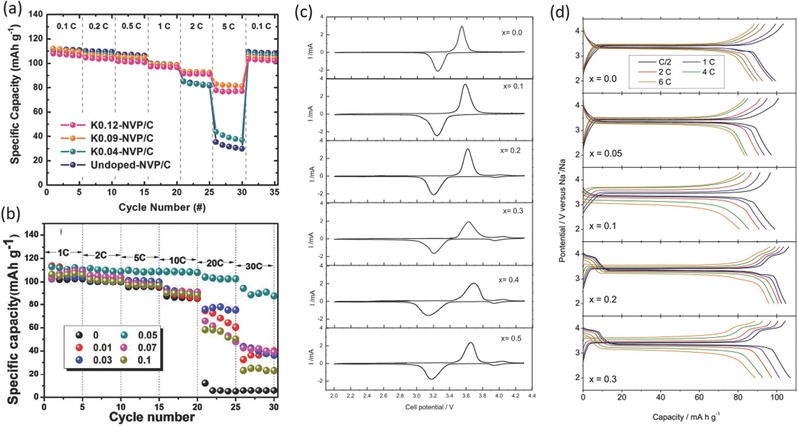
(a) Rate capabilities (0.1–5 C) of Na_3−_ *_x_*K*_x_*V_2_(PO_4_)_3_/C; (b) Rate capability of Na_3_V_2–x_Mg_x_(PO_4_)_3_/C (x = 0, 0.01, 0.03, 0.05, 0.07 and 0.1) at different current densities; (c) Cyclic voltammograms of the Na_3_V_2–x_Fe_x_(PO_4_)_3_ series recorded at a scan rate of 0.5 mV s^–1^; (d) Galvanostatic charge and discharge curves of Na_3_V_2–x_Al_x_(PO_4_)_3_ samples performed at several C rates. (a) Reproduced with permission.^81^ Copyright 2014, Royal Society of Chemistry. (b) Reproduced with permission.^82^ Copyright 2015, Royal Society of Chemistry. (c) Reproduced with permission.^84^ Copyright 2015, The Electrochemical Society. (d) Reproduced with permission.^87^ Copyright 2015, Elsevier.

Overall, the promising results above‐mentioned have ignited tremendous efforts to improve the electrochemical performance of the Na_3_V_2_(PO_4_)_3_ electrode, including metal ion doping, carbon coating and particle downsizing. Metal ion doping was widely conducted to improve the structural stability of the Na_3_V_2_(PO_4_)_3_. K‐ions with larger ionic radius were incorporated as functional pillar ions into the Na_3_V_2_(PO_4_)_3_ structure by Kim et al. These results indicate that the K‐ions play an important role in enlarging the Na‐ion diffusion pathway and elongating the *c*‐axis thus to increase the lattice volume.^81^ Na_3_V_2−_ *_x_*Mg*_x_*(PO_4_)_3_/C composites with various Mg^2+^ doping contents were investigated by Li et al.^82^ The doped Mg was substituted for the vanadium site and did not alter the structure. The ionic and electronic conductivities of the Na_3_V_2−_ *_x_*Mg*_x_*(PO_4_)_3_ are significantly improved after Mg doping, resulting in a enhancement of the rate and cycle performances (**Figure** **5**b).^82^, ^83^ Lavela's group has done extensive work about various metal ions substitution.^84^, ^85^, ^86^, ^87^ Iron substitution (both Fe^2+^ and Fe^3+^) was also found to effectively activate a V^4+/5+^ redox couple. In addition, the cell volume was increased after the larger Fe^3+^ substitution, resulting in the distortion of M1 octahedra, thus Na ions residing at M1 sites can be extracted for electrochemical reaction (Figure 5c).^84^, ^88^, ^89^ Similar phenomenon was also found by chromium, manganese and aluminum substitution (Figure 5d), respectively, by the same group.^85^, ^86^, ^87^

Carbon decoration has been confirmed to be an effect strategy to improve the electrochemical performance of the Na_3_V_2_(PO_4_)_3_ electrode and many research groups employed different carbon matrixes to prepare high‐performance electrodes.^90^, ^91^, ^92^, ^93^, ^94^, ^95^, ^96^, ^97^, ^98^ Duan et al. synthesized Na_3_V_2_(PO_4_)_3_@C core‐shell nanocomposite by hydrothermal assisted sol‐gel method and obtained an initial capacity of 104.3 mAh g^−1^ at 0.5 C and 94.9 mAh g^−1^ at 5 C with a remarkable capacity retention of 96.1% after 700 cycles.^99^ Mechanically ball milling methods, including low temperature pre‐reduction and high temperature carbon thermal reduction, were reported to construct carbon coated Na_3_V_2_(PO_4_)_3_ electrodes with improved performance.^100^, ^101^, ^102^ Nitrogen and Boron doped carbon were also used to coat the Na_3_V_2_(PO_4_)_3_, respectively.^103^, ^104^, ^105^ The Boron doping can increase numerous extrinsic defects and active sites in the carbon coated layer, which could significantly accelerate Na^+^ transport in the carbon layer thus to greatly improve the rate performance and cycling stability of the Na_3_V_2_(PO_4_)_3_/C+B electrode.^104^ Encapsulating Na_3_V_2_(PO_4_)_3_ nanoparticles in one‐dimensional carbon sheath were widely reported through an electrospinning method by different groups.^106^, ^107^, ^108^ The one‐dimensional sodium ion transport pathway and the highly conductive network can further improve the electrochemical performance greatly. Due to its remarkably high conductivity and mechanical properties, graphene was also introduced to improve the performance of the Na_3_V_2_(PO_4_)_3_ electrode,^12^, ^83^, ^105^, ^109^, ^110^, ^111^, ^112^, ^113^ for example, Jung and Tao et al. have reported a graphene‐supported Na_3_V_2_(PO_4_)_3_ with a rate capability of 30 C and a stable cycling performance over 300 cycles.^109^, ^110^

**Figure 6:**
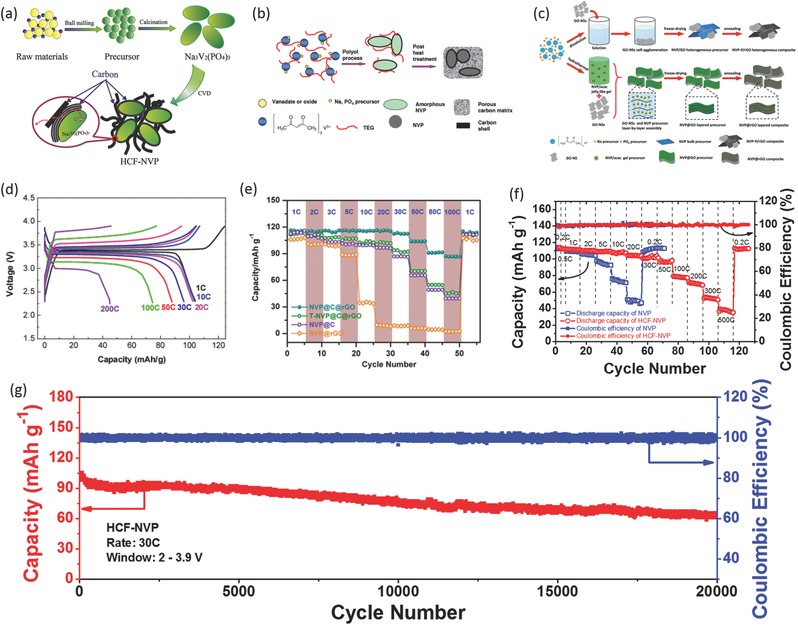
(a) Schematic illustration of the synthesis of hierarchically carbon coated Na_3_V_2_(PO_4_)_3_; (b) Facile softchemistry‐based double carbon‐embedding approach for (C@NVP)@pC; (c) Schematic illustration for the synthesis of NVP‐P/GO and NVP@rGO composites; (d) Galvanostatic charging−discharging profiles of (C@NVP)@pC at different current rates; (e) Rate performance of NVP@C@rGO, T‐NVP@C@rGO, NVP@C, and NVP@rGO cathodes; (f) Rate capability of the NVP and HCF‐NVP electrodes; (g) Long‐term cycling performance of the HCF‐NVP electrode at a high current rate of 30 C over 20 000 cycles. (a,f,g) Reproduced with permission.^11^ Copyright 2015, Wiley‐VCH. (b,d) Reproduced with permission.^114^ Copyright 2014, American Chemical Society. (e) Reproduced with permission.^12^ Copyright 2015, Wiley‐VCH.

Recently, the use of hierarchical carbon to decorate the Na_3_V_2_(PO_4_)_3_ electrode has been widely adopted to make high‐performance Na_3_V_2_(PO_4_)_3_ electrodes.^11^, ^12^, ^60^, ^105^, ^111^, ^112^, ^113^, ^114^, ^115^, ^116^ Zhu et al. reported a carbon‐coated Na_3_V_2_(PO_4_)_3_ embedded in porous carbon with remarkable rate capability of 44 mAh g^–1^ at a current rate of 200 C (**Figure** **6**d).^114^ Xu et al. have also synthesized a layer‐by‐layer Na_3_V_2_(PO_4_)_3_@rGO material with ≈3% rGO and 0.5% amorphous carbon, which shows excellent rate capability (41 mAh g^–1^ at a current rate of 200 C) and cyclability (70.0% capacity retention after 15 000 cycles at 50 C).^112^ Coated carbon formed from pyrolysis of organic precursor is usually in an amorphous state with low electric conductivity. To further improve the electron conductivity of Na_3_V_2_(PO_4_)_3_, Fang et al. reported a facile chemical vapor deposition (CVD) method to build highly conductive coated‐carbon with graphical structure. The hierarchical carbon framework‐wrapped Na_3_V_2_(PO_4_)_3_ exhibited an unprecedented electrochemical performance of both ultra‐high rate capability (38 mA h g^–1^ at 500 C) and ultra‐long cycling stability (54% capacity retention over 20 000 cycles at 30 C rate) (Figure 6f,g).^11^ The highly conductive carbon framework is strongly effective in promoting ultra‐fast electronic transport and assisting in buffering volume change during Na ion insertion/deinsertion.

Apart from being used as cathode material, the Na_3_V_2_(PO_4_)_3_ was also widely investigated as anode materials due to the lower potential of V^2+/3+^ an V^1+/2+^ redox couple.^117^, ^118^, ^119^ The Na_3_V_2_(PO_4_)_3_ anode could reversibly insert/extract three sodium ions between 3.0 and 0.01 V, corresponding to a reversible capacity of about 170 mAh g^–1^, and exhibited two voltage plateaus at 1.57 and 0.28 V.^117^, ^118^ Based on the low potential of Na_3_V_2_(PO_4_)_3_, many symmetric cells constructed of Na_3_V_2_(PO_4_)_3_ electrodes were reported.^72^, ^90^, ^113^, ^120^, ^121^ For example, Zhang et al. have assembled a symmetric full cell by using self‐supporting Na_3_V_2_(PO_4_)_3_ electrodes, the full cell exhibited an output voltage plateau of 1.8 V with a capacity of 90.2 mAh g^–1^ and a 81% capacity retention over 280 cycles.^120^

#### 2.2.2 NASICON‐type NaTi_2_(PO_4_)_3_

NaTi_2_(PO_4_)_3_ was first reported by Hagman et al.^122^ This compound possesses a NASICON crystalline structure similar to Na_3_V_2_(PO_4_)_3_ with a rhombohedral structure in the R‐3c space group and two different Na sites, where M2 sites can reversibly insert two Na^+^, corresponding a theoretical capacity of 133 mAh g^–1^. Delmas et al. first studied the Na storage behavior of NaTi_2_(PO_4_)_3_, and found that two Na ions could reversibly insert in NaTi_2_(PO_4_)_3_ at room temperature either chemically and electrochemically.^123^ The NaTi_2_(PO_4_)_3_ electrode exhibited a discharge plateau at 2.1 V (vs. Na/Na^+^), associated with a two‐phase reaction. The moderate voltage range ensures that the NaTi_2_(PO_4_)_3_ can be used as anode and cathode depending on the counter electrodes. When used as anodes, the NaTi_2_(PO_4_)_3_ exhibits a higher coulombic efficiency with much higher safety due to the reduction of the possibility of forming a solid electrolyte interface (SEI) and avoidance of sodium deposition. The moderate voltage range and stable structure also make it an idea candidate as anode materials in aqueous solution, and the studies of NaTi_2_(PO_4_)_3_ in aqueous solution will be discussed in section 5.1.

**Figure 7:**
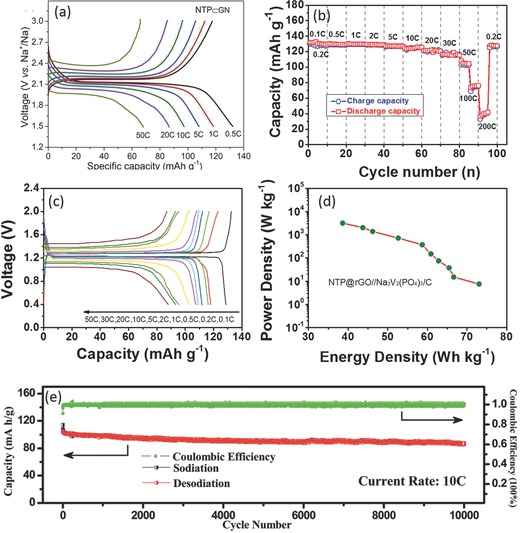
(a) Galvanostatic discharge–charge profiles of the NTP‐GN electrode at various current rates; (b) Rate capability of the NTP@rGO electrode; (c) Rate capability of the NTP@rGO//Na_3_V_2_(PO_4_)_3_/C sodium ion battery; (d) Ragone plots of the NTP@rGO//Na_3_V_2_(PO_4_)_3_/C sodium ion battery based on the cathode and anode mass; (e) Cycling performances of the NTP‐NBA electrodes obtained at 10 C rate. (a) Reproduced with permission.^130^ Copyright 2015, American Chemical Society. (b,c,d) Reproduced with permission.^135^ Copyright 2016, Wiley‐VCH. (e) Reproduced with permission.^129^ Copyright 2015, Royal Society of Chemistry.

Due to the intrinsic low electronic conductivity of the phosphate framework, improving the electron conductivity of the NaTi_2_(PO_4_)_3_ material by carbon matrix becomes an important route to obtain electrodes with high‐performance.^124^, ^125^, ^126^, ^127^, ^128^, ^129^, ^130^, ^131^, ^132^, ^133^ NaTi_2_(PO_4_)_3_ nanoparticles were synthesized by many groups through solvothermal strategy with the aim at promoting the electrode performance.^124^, ^125^, ^126^, ^127^, ^128^, ^129^, ^130^ For example, Wu and Wang et al. reported grapheme‐ and carbon nanotube‐ decorated NaTi_2_(PO_4_)_3_ nanoparticles, respectively, both of which had a high rate capability of 50 C rate (**Figure** **7**a) and long cycle life of 1000 cycles.^127^, ^130^ Yang et al. have synthesized porous NaTi_2_(PO_4_)_3_ nanocubes with controllable size and the as‐prepared products have shown outstanding high‐rate capability of 100 C with long cycling life of 10 000 cycles (Figure 7e).^129^ Carbon‐ and rutile TiO_2_‐coated NaTi_2_(PO_4_)_3_ nanocubes were reported by Yang et al., the composite also exhibited high cycle stability with capacity retention of 89.3% over 10 000 cycles.^126^ Hierarchical carbon matrixes were also reported to effectively improve the electrochemical performance of the NaTi_2_(PO_4_)_3_ electrode. Jiang et al. have synthesized thinner carbon shell and interconnected carbon network decorated NaTi_2_(PO_4_)_3_, which showed high reversible capacity of 108 mAh g^–1^ at 100 C and a long cycle life of 83 mAh g^–1^ at 50 C after 6000 cycles.^134^ Fang et al. also reported a spray‐drying method to prepare hierarchical graphene supported NaTi_2_(PO_4_)_3_, where graphene coated nanosized NaTi_2_(PO_4_)_3_ and 3D graphene network could be achieved simultaneously. The electrode exhibited high reversible capacity of 130 mAh g^–1^ at 0.1 C and an ultra‐high rate capability of 38 mAh g^–1^ at 200 C (Figure 7b). Simultaneously, an all NASICON‐type NaTi_2_(PO_4_)_3_//Na_3_V_2_(PO_4_)_3_ full cell was also assembled with superior capacity and high‐power performance (Figure 7c and d).^135^ The structural elucidation during electrochemical reaction was studied by density functional theory on a full‐titanium‐base symmetric cell.^136^ The Na storage at low operation voltages was also found to associate with an extra working plateau at around 0.4 V,^137^, ^138^ which was attributed to the further reduction of Ti^3+^ to Ti^2+^. The electrode could also give a huge improved reversible capacity of ≈210 mAh g^–1^ at 0.1 C, a rate capability of 50 mAh g^–1^ at 100 C and long‐term cycling life with ≈68% capacity retention over 10 000 cycles.^137^

Iron‐substituted NaTi_2_(PO_4_)_3_ was investigated by a number of groups.^139^, ^140^, ^141^ Differing from NaTi_2_(PO_4_)_3_, the Fe‐substituted sample, Na_1.5_Fe_0.5_Ti_1.5_(PO_4_)_3_, exhibited more complex mechanisms than the two‐phase and one‐phase mechanisms observed for NaTi_2_(PO_4_)_3_ and Na_3_Fe_2_(PO_4_)_3_, respectively, and iron was an electrochemically active center at 2.2 V with the reversible Fe^3+^/Fe^2+^ transformation.^140^, ^141^

### 2.3 Amorphous Phosphate Structure

Amorphous materials lacks the long‐range ordering of a crystal but has certain short‐range ordering at atomic length scale due to their favorable chemical bonding.^142^, ^143^ Due to the lack of three‐dimensional long‐range order, amorphous solids do not constructively diffract X‐rays, as do crystalline solids. Therefore, broad, diffuse haloes are observed in X‐ray powder diffraction patterns instead of well‐defined peaks.^143^ Amorphous solids are supposed as potential electrodes considering less lattice pressure during electrochemical reaction. However, it is not easy to make an amorphous structure for most of the crystalline electrode materials with satisfying electrochemical performance because of the metastable state of amorphous materials.

**Figure 8:**
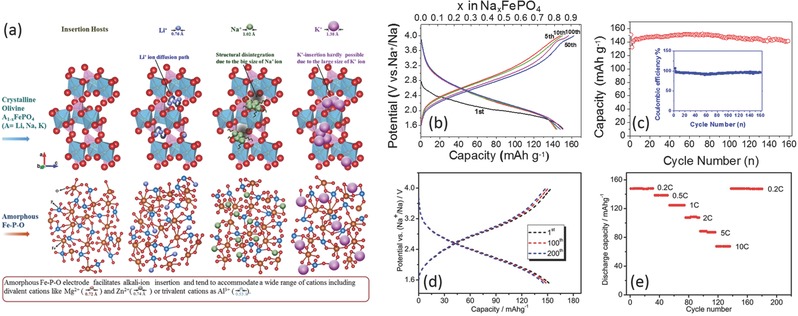
(a) Schematic representation of alkali‐ions (Li/Na/K) insertion in crystalline and amorphous FePO_4_ electrode hosts; (b,c) Galvanostatic discharging/charging profiles of the FePO_4_/C cathode performed at a current density of 20 mA g^–1^ and the corresponding cycling performance; (d,e) Galvanostatic discharging‐charging profiles performed at a current density of 0.1 C and rate capability of amorphous NaFePO_4_ nanospheres. (a) Reproduced with permission.^145^ Copyright 2014, Nature Publishing Group. (b,c) Reproduced with permission.^10^ Copyright 2014, American Chemical Society. (d,e) Reproduced with permission.^156^ Copyright 2015, Royal Society of Chemistry.

Among the phosphate compounds, iron‐based phosphates are easy to form amorphous phase. Amorphous FePO_4_ has been widely reported as cathodes for lithium ion batteries with high reversible capacity (175 mAh g^–1^) and stable cyclability, and was investigated as drop‐in replacements for SIBs. Shiratsuchi et al. firstly compared the Li and Na storage performance of amorphous and crystalline FePO_4_ and found that both amorphous and crystalline FePO_4_ showed similarly reversible capacities not only for Li but also for Na ion batteries. The amorphous FePO_4_ for Na ion storage delivered an optimal capacity of 146 mAh g^–1^ at a current rate of 0.1 mA cm^–2^.^144^ Mathew et al. have studied the amorphous FePO_4_ as host for various charge carrier ions (mono‐/di‐/tri‐valent ions) (**Figure** **8**a), which delivered a capacity of 179 mAh g^–1^ for SIBs, based on a reversible amorphous‐to‐crystalline transition during electrochemical reactions.^145^ Zhao et al. used a solvent extraction route to obtain monodisperse amorphous FePO_4_·2H_2_O nanospheres, which exhibited a reversible Na ion storage capacity of 108 mAh g^–1^ at 3.5 mA g^–1^.^146^

Because high temperature calcination can lead to the crystallization of FePO_4_, carbon decoration of this material has to be conducted at a relative low temperature. Various carbon matrixes have been introduced to improve the Na storage performance of the amorphous FePO_4_. Liu et al. reported single‐wall carbon nanotubes wired FePO_4_ with reversible capacity of 120 mAh g^–1^.^147^ Fang et al. also reported a mesoporous amorphous FePO_4_ embedded in carbon matrix. The obtained FePO_4_/C exhibited a high initial discharge capacity of 151 mAh g^–1^ at 20 mA g^–1^ with stable cyclability (94% capacity retention ratio over 160 cycles) (Figure 8b,c) as well as high rate capability (44 mAh g^–1^ at 1000 mA g^–1^), and the electrode kept amorphous structure at different states of charge.^10^ Multi‐walled carbon nanotubes, graphene and carbonized polyaniline decorated FePO_4_ were also synthesized with well‐improved electrochemical performances.^148^, ^149^, ^150^, ^151^ The Na storage behavior of amorphous and crystalline FePO_4_ were compared by Wang and Liu et al.^152^, ^153^ The amorphous FePO_4_ have been testified to exhibit better reversible capacity and cycling stability over the crystalline FePO_4_.^152^, ^153^ During sodiation process, the amorphous FePO_4_ transformed into NaFePO_4_ with amorphous and triphylite phase simultaneously, while trigonal FePO_4_ partly transformed into the maricite NaFePO_4_.^153^, ^154^ Recently, 2D amorphous iron phosphate nanosheets were also reported to have a high initial discharge capacity of 168.9 mA h g^−1^ at 0.1 C and a stable cycle life with 92.3% capacity retention over 1000 cycles, showing a highest reversible capacity among phosphate framework materials.^155^

The above discussed FePO_4_ are Na‐vacant, which is not convenient for practical battery applications as cathode materials. Recently, Li et al. reported Na‐riched, amorphous NaFePO_4_ nanospheres with a high initial discharge capacity of 152.1 mAh g^–1^ (Figure 8d), high rate capability (67.4 mAh g^–1^ at 10 C) (Figure 8e) and stable cyclability (95% capacity retention over 300 cycles).^156^ More Na‐rich amorphous materials are needed to explore for enriching the amorphous cathode systems.

## 3 Pyrophosphate Materials for Na Storage

In parallel to the massive effort on phosphate materials, their analogue, pyrophosphate materials, have also attracted wide interest. The sodium metal pyrophosphate compounds generally consist of transition metal octahedral MO_6_ and P_2_O_7_ units connected to form a robust framework.^157^

**Figure 9:**
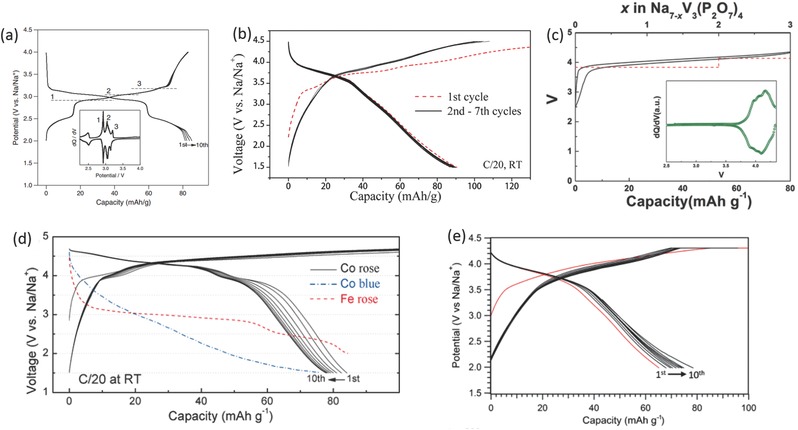
(a) Galvanostatic voltage‐composition curve of Na_2_FeP_2_O_7_ at a rate of C/20; (b) Galvanostatic charge‐discharge curves of Na_2_MnP_2_O_7_; (c) A charge/discharge profile of Na_7_V_3_(P_2_O_7_)_4_ with the calculated voltage (inset: d*Q*/d*V* of Na_7_V_3_(P_2_O_7_)_4_); (d) Galvanostatic cycles of the Co rose (RN), Fe rose, and Co blue polymorphs; (e) Voltage‐capacity charge‐discharge profile of Na_2_(VO)P_2_O_7_ cathode at a rate of C/20. (a) Reproduced with permission.^158^ Copyright 2012, Elsevier. (b) Reproduced with permission.^176^ Copyright 2013, American Chemical Society. (c) Reproduced with permission.^184^ Copyright 2016, Wiley‐VCH. (d) Reproduced with permission.^180^ Copyright 2016, Wiley‐VCH. (e) Reproduced with permission.^183^ Copyright 2014, Wiley‐VCH.

Komatsu and Yamada's group first reported the pyrophosphate‐class material for SIBs. They first tested a Na_2_FeP_2_O_7_ material to obtain a reversible capacity of 82 mAh g^–1^ with the Fe^3+^/Fe^2+^ redox potential at around 3 V and 2.5 V (**Figure** **9**a).^158^, ^159^ It is found that the Na_2_FeP_2_O_7_ phase and desodiated NaFeP_2_O_7_ phase delivered excellent thermal stability up to 600 °C with no thermal decomposition and/or oxygen evolution due to the stable pyrophosphate (P_2_O_7_)^4–^ anion.^160^ Atomistic simulation indicated that the Na_2_FeP_2_O_7_ exhibited a 3D Na^+^ diffusion behavior.^161^ Kim et al. also conducted combined experimental and theoretical study to investigate the structure, electrochemical and thermal properties of Na_2_FeP_2_O_7_ material. Both quasi‐equilibrium measurements and first‐principles calculations consistently indicated that Na_2_FeP_2_O_7_ underwent two kinds of reactions: a single‐phase reaction around 2.5 V and a series of two‐phase reactions in the voltage range of 3.0–3.25 V.^162^ After that, some efforts were made to optimize the electrochemical performance of Na_2_FeP_2_O_7_ electrode for SIBs.^163^, ^164^, ^165^, ^166^, ^167^, ^168^ For example, carbon nanotubes decorated Na_2_FeP_2_O_7_ was demonstrated to have a high rate capability at 20 C rate,^166^ and can work well in inorganic ionic liquid.^167^, ^168^ Due to the higher electrochemically active Mn^3+^/Mn^2+^ redox potential, Manganese substitution were also employed to raise the average redox potential up to 3.2 V.^169^, ^170^ Ex situ XRD and CV analyses indicated that Na_2_Fe_0.5_Mn_0.5_P_2_O_7_ underwent a single phase reaction rather than a biphasic reaction due to different Na coordination environment and different Na sites occupancy.^169^ Recently, off‐stoichiometric iron‐based pyrophosphate, named Na_2–x_Fe_1+x/2_P_2_O_7_, were reported To have similar structure and charge/discharge plateaus with Na_2_FeP_2_O_7_, but higher reversible capacity (114 mAh g^–1^) and better cyclability exceeding 3000 cycles in ionic liquid electrolyte.^171^, ^172^, ^173^, ^174^, ^175^

Na_2_MnP_2_O_7_ was also introduced as electrode materials for SIBs,^176^, ^177^ exhibiting good electrochemical activity at ≈3.8 V with reversible capacity of 90 mAh g^–1^ (Figure 9b). First‐principles calculations indicated that the enhanced electrochemical performance was mainly resulted from the small extent of atomic rearrangements, which lower the barriers for electron conduction and phase boundary migration.^176^

In addition to Na_2_MnP_2_O_7_, Na_2_CoP_2_O_7_ was also investigated as a high voltage cathode.^178^, ^179^, ^180^ Yamada's group reported an orthorhombic structure Na_2_CoP_2_O_7_ with the space group of *Pna*2_1_, which offered a two‐dimensional Na‐diffusion pathway and could deliver a reversible discharge capacity of 80 mAh g^–1^ at an average potential of 3 V.^178^ Kim et al. have employed a strategy by controlling the Na deficiencies to successfully obtain a triclinic Na_2_CoP_2_O_7_, which showed great improvement of energy density. The optimized material showed an average voltage of 4.3 V with reversible capacity of 80 mAh g^–1^ (Figure 9d).^180^ This work may provide a concept of developing new materials via nonstoichiometry‐driven control of polymorphism.

Vanadium‐based pyrophosphates exhibit structural diversity with different compositions, such as NaVP_2_O_7_, Na_7_V_3_(P_2_O_7_)_4_, and *t*‐Na_2_(VO)P_2_O_7_.^181^, ^182^, ^183^, ^184^ The *t*‐Na_2_(VO)P_2_O_7_ delivered a capacity of 80 mAh g^–1^ through V^5+^/V^4+^ redox reaction at the potential of 3.8 V (Figure 9e),^183^ while Na_7_V_3_(P_2_O_7_)_4_ showed a capacity of 80 mAh g^–1^ with average potential of 4 V (Figure 9c).^184^ The high‐voltage vanadium‐based pyrophosphates paved the way for further exploration of Na_2_MP_2_O_7_ family.

## 4 Mixed‐Anion Materials for Na Storage

A series of compounds with new classes of host structures and compositions can be obtained by using mixed‐anion approach. By introducing new anion into the phosphate structure, some of the mix‐anion compounds can show improved electrochemical performance. For example, the F‐substituted sample exhibited higher potential due to the strong inductive effect of the F^−^ anion. In the phosphate materials with mixed‐anion structure, F‐substituted samples and mixed multivalent anions (CO_3_^2–^, P_2_O_7_^4–^) substituted samples showed better electrochemical performance.

### 4.1 F‐substituted Materials for Na Storage

The combination of F anions with phosphates will lead to a variety of compounds with enhanced operating voltage due to the higher iconicity of the M—F bond.^185^ Among these materials, the Fe‐based and V‐based materials emerged to have excellent Na storage performance.

#### 4.1.1 Fe‐based F‐substituted Materials

**Figure 10:**
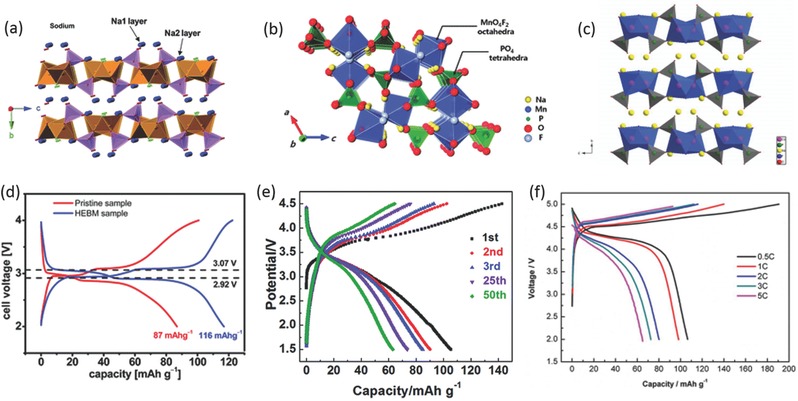
View of the crystal structure of (a) Na_2_FePO_4_F with *Pbcn* space group, (b) Na_2_MnPO_4_F with *P*2_1_/*n* space group, and (c) Na_2_CoPO_4_F with *Pbcn* space group; Galvanostatic charge‐discharge curves of the (d) Na_2_FePO_4_F pristine and ball milled samples, (e) Na_2_MnPO_4_F sample, and (f) Na_2_CoPO_4_F/C sample. (a,d) Reproduced with permission.^192^ Copyright 2015, Royal Society of Chemistry. (b) Reproduced with permission.^195^ Copyright 2012, Royal Society of Chemistry. (c,f) Reproduced with permission.^199^ Copyright 2015, The Electrochemical Society. (e) Reproduced with permission.^196^ Copyright 2014, Royal Society of Chemistry.

Na_2_FePO_4_F was first employed as cathode for lithium ion batteries by Nazar's group. This material has a layered structure with a *Pbcn* orthrorhombic space group, in which face‐sharing FeO_4_F_2_ octahedra are connected via bridging F atoms to form chains and are joined by PO_4_ tetrahedra to form [FePO_4_F] infinite layers with Na cations located in the interlayer space (**Figure** **10**a).^186^, ^187^ Tarascon's group first tested Na_2_FePO_4_F for SIBs, which exhibited two well‐defined discharge voltage plateaus at 3.1 and 2.9 V with a reversible capacity of 0.8 Na per unit formula.^188^ Enhanced electrochemical performance has been reported by many groups through carbon coating technology and different synthesis routes.^189^, ^190^, ^191^, ^192^ For example, Law et al. have synthesized Na_2_FePO_4_F via a soft template method to deliver impressive capacity of 116 mAh g^–1^ (Figure 10d), high rate capability of 21 mAh g^–1^ at 10 C rate and 80% capacity retention over 200 cycles.^192^ Atomistic simulation method was introduced to study the Na ion migration property of Na_2_FePO_4_F. Na ion conduction in Na_2_FePO_4_F was predicted to be two‐dimensional (2D) in the interlayer plane with a low activation energy, indicating high Na mobility through a 2D network in the *ac* plane.^193^ Manganese substitution was also reported, due to the Mn^2+^/Mn^3+^ redox reaction.^188^, ^194^ The manganese substituted sample exhibited higher average operating voltage, which was sufficient to trigger a 2D–3D structural transition.^188^ The reported Na_2_MnPO_4_F possesses a three‐dimensional *P*2_1_/*n* structure (Figure 10b) with sloping charge/discharge curves (Figure 10e). First principles calculations indicated that extracting the second Na ion from Na_2_MnPO_4_F required a much higher voltage (≈4.67 V vs. Na/Na^+^).^195^, ^196^, ^197^ The Na_2_CoPO_4_F also has a two‐dimensional layered structure (Figure 10c), which exhibited high discharge voltage of 4.3 V with reversible capacity of ≈ 100 mAh g^–1^. However, this material showed a low coulombic efficiency with degenerated capacity (Figure 10f).^198^, ^199^

#### 4.1.2 V‐based F‐substituted Materials

F‐substitution can enrich largely the family of V‐based materials and introduce new structure with varying electrochemical properties. The inductive effect of F anions can also elevate the operating voltage. With respect to sodium‐vanadium fluorophosphates, three phases widely investigated are NaVPO_4_F, Na_3_V_2_(PO_4_)_2_F_3_ and Oxygen substituted Na_3_(VO_1−_ *_x_*PO_4_)_2_F_1+2_ *_x_* (0 ≤ *x* < 1).

NaVPO_4_F was first reported by Barker et al.^200^ They assembled a hard carbon//NaVPO_4_F full cell, which exhibited an average working potential of 3.6 V with reversible capacity of about 80 mAh g^–1^. The effect of carbon and graphene coating on NaVPO_4_F was also reported.^201^, ^202^ Cr and Al substitution were reported to be effective for the cycle stability.^203^, ^204^ However, it's worth noting that there are no structural data about the NaVPO_4_F phase. The XRD data shown in these works match well with the NASICON compound, so that the existence of the NaVPO_4_F phase has been questioned by some authors.^205^, ^206^

**Figure 11:**
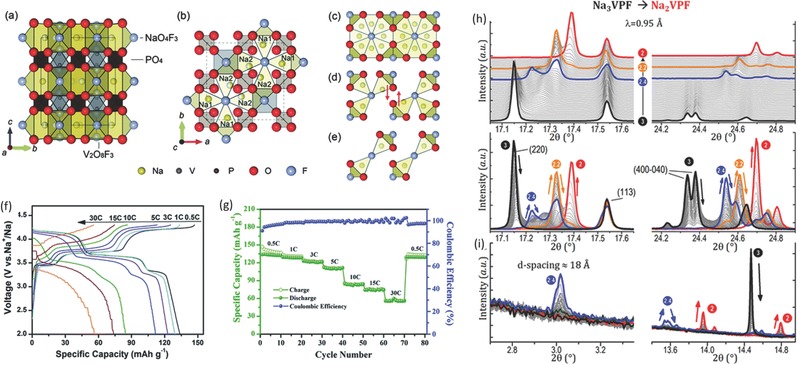
Schematic representation of a refined Na_3_V_2_(PO_4_)_2_F_3_ structure projected along (a) the *a* axis and (b) the *c* axis, (c) all of the possible Na sites and (d) most stable configuration of Na ions in Na_3_V_2_(PO_4_)_2_F_3_ and (e) Na_2_V_2_(PO_4_)_2_F_3_ from the first principles calculations. Na1 indicates fully occupied Na sites and Na2 indicates half occupied Na sites. Red arrows of (d) represent a shift of Na ions off the centers of prismatic sites. (f) Galvanostatic charging‐discharging profiles of the NVPF@C nanocomposite at various current rates; (g) Rate capability at various current rates of the NVPF@C nanocomposite. Different angular domains observed during the extraction of 1 Na^+^ from Na_3_VPF (black curve). Several single‐phase compositions are formed through biphasic domains, namely, Na_2.4_VPF (blue), Na_2.2_VPF (orange), and Na_2_VPF (red). (h) 17°–17.7° (left) and 24.1°–24.9° (right) angular domains. Peaks are indexed for the Na_3_VPF phase. (i) 2.7°–3.3° and 13.5°–14.9° angular domains (weak peaks related to sodium ordering). (a–e) Reproduced with permission.^211^ Copyright 2012, Royal Society of Chemistry. (f,g) Reproduced with permission.^213^ Copyright 2015, Royal Society of Chemistry. (h,i) Reproduced with permission.^214^ Copyright 2015, American Chemical Society.

The similar structure, Na_3_V_2_(PO_4_)_2_F_3_, is attracting strong interest as cathodes for SIBs due to its high capacity, rate capability and long‐term cycling stability. Meins et al. first reported the crystal structure of Na_3_V_2_(PO_4_)_2_F_3_ with a tetragonal structure (*P4_2_/mnm* space group) (**Figure** **11**a–e),^207^ featuring a strongly covalent 3D framework with large interstitial spaces for ion diffusion. Not only the specially constructed [PO_4_]^3–^ network can help to stabilize the crystal structure of the material, but the oxygen atoms fixed in the [PO_4_]^3–^ formation may also decrease the likelihood of oxygen liberation, leading to better thermal stability.^208^ Barker's group first studied lithium storage performance of Na_3_V_2_(PO_4_)_2_F_3_.^209^, ^210^ Shakoor et al. studied the Na storage performance of Na_3_V_2_(PO_4_)_2_F_3_ through combined computation and experiments. This material exhibited two plateaus with average voltages of about 3.7 and 4.2 V. Structural evaluation indicated that the reversible sodiation/desodiation occured through one‐phase reaction.^211^ High‐performance Na_3_V_2_(PO_4_)_2_F_3_ electrodes were also reported.^212^, ^213^ For example, Liu et al. have synthesized carbon coated Na_3_V_2_(PO_4_)_2_F_3_, which delivered a high reversible capacity of 130 mAh g^−1^ with high rate capabitlity (57 mAh g^–1^ at 30 C rate) (Figure 11f,g) and long cycle life (50% capacity retention over 3000 cycles).^213^ The Na ion (de)intercalation mechanism was well studied by some groups.^208^, ^214^, ^215^, ^216^ Bianchini et al. have performed high angular resolution synchrotron radiation diffraction measurement to carefully reveal the phase diagram. It was found that four intermediate phases existed during the Na extraction reaction and only one of these phases underwent a solid solution reaction (Figure 11h,i).^214^ Liu et al. studied the structural and dynamical changes of Na_3_V_2_(PO_4_)_2_F_3_ during charge process and found distinct changes in Na‐ion and electronic mobility and V electronic configurations: the Na ions were removed non‐selectively from the two distinct Na sites, while Na mobility increased steadily with increased more Na vacancies in the structure on charging.^216^

**Figure 12:**
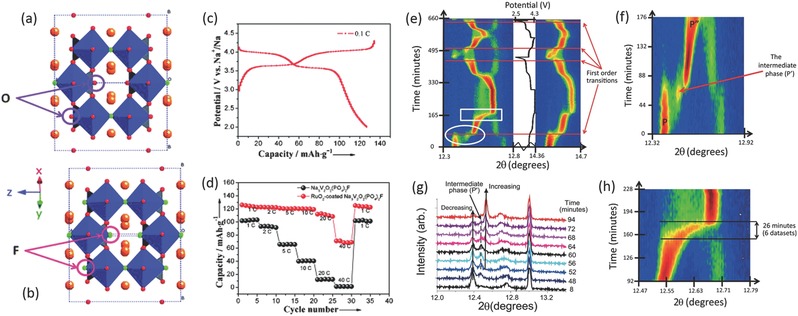
Structural comparison of (a) Na_3_(VO)_2_(PO_4_)_2_F and (b) Na_3_V_2_(PO_4_)_2_F_3_; (c) Charge and discharge profiles of RuO_2_‐coated Na_3_V_2_O_2_(PO_4_)_2_F nanowires at a current density of 0.1 C; (d) Rate capacity of RuO_2_‐coated Na_3_V_2_O_2_(PO_4_)_2_F nanowires and uncoated Na_3_V_2_O_2_(PO_4_)_2_F nanowires; Evidence of reaction mechanism evolution of the cathode during charge and discharge. (e) Selected 2θ regions of in situ synchrotron XRD data of Na_3_V_2_O_2_(PO_4_)_2_F highlighting the evolution of the 220, 113 (left) and 311, 222 (right) reflections by a color scale and the potential profile. Selected temporal region (f) and snapshots (g) of the in situ data of the 220 and 113 reflections with the phases P, P′, and P″ shown. (h) Selected temporal region of in situ data showing the two‐phase region at higher charge. (a,b) Reproduced with permission.^205^ Copyright 2012, Royal Society of Chemistry. (c,d) Reproduced with permission.^225^ Copyright 2015, Wiley‐VCH. (e–h) Reproduced with permission.^223^ Copyright 2014, American Chemical Society.

A family of oxygen substituted samples, namely Na_3_(VO_1−_ *_x_*PO_4_)_2_F_1+2_ *_x_* (0 ≤ *x* < 1), have attracted much attention due to the high energy density and good cycle life.^205^, ^217^, ^218^, ^219^, ^220^, ^221^ In all cases, the samples have similar X‐ray diffraction characteristics and charge/discharge curves with two voltage plateaus at the same voltages, suggesting that all the materials belong to the same family of compounds, where the fluorine content is modulated by the presence of V^3+^ and VO^2+^ (V^4+^) and the redox mechanism varies depending on the compositions.^205^ An example of structural comparison of Na_3_V_2_(PO_4_)_2_F_3_ and Na_3_(VO)_2_(PO_4_)_2_F phases is shown in **Figure** **12**a–b. Both of them present the same framework, where one of the fluorine atoms in Na_3_V_2_(PO_4_)_2_F_3_ is replaced by an oxygen in Na_3_(VO)_2_(PO_4_)_2_F.^205^ Teófilo Rojo's group have done much work to study the Na storage mechanism of the Na_3_(VO_1−_ *_x_*PO_4_)_2_F_1+2_ *_x_* materials.^220^, ^222^, ^223^ The reaction mechanisms during charge/discharge process include combinations of solid solution and two‐phase reaction behavior, but the structural motif was maintained throughout these reactions.^223^ The relationship among V^3+^/V^4+^/V^5+^ redox reactions, Na^+^−Na^+^ ordering, and Na^+^ intercalation mechanisms of Na_3_(VO_1−_ *_x_*PO_4_)_2_F_1+2_ *_x_* in SIBs were investigated by Park et al. through a combined theoretical and experimental approach.^218^, ^224^ They found that the redox mechanism and phase reactions varied with fluorine content. High performance Na_3_(VO_1−_ *_x_*PO_4_)_2_F_1+2_ *_x_* electrodes were also reported. Qi et al. have synthesized a series of Na_3_(VO_1−_ *_x_*PO_4_)_2_F_1+2_ *_x_* (0 ≤ *x* ≤1) materials by a solvothermal strategy. Among them, the Na_3_(VOPO_4_)_2_F sample exhibited the best Na‐storage performance with both high rate capability and long cycle life.^219^ Peng et al. reported a RuO_2_‐coated Na_3_V_2_O_2_(PO_4_)_2_F nanowires with enhanced electrochemical performance of high reversible capacity of 120 mAh g^–1^, high rate capability (95 mAh g^–1^ at 20 C rate) (Figure 12c–d) and long cycle life of 1000 cycles.^225^

### 4.2 Mixed Multivalent Anion Materials for Na Storage

Combining different multivalent anions together, we can get a series of materials with novel structure and electrochemical activity. The compounds containing (PO_4_)(P_2_O_7_) and (PO_4_)(CO_3_) are in the spotlight due to the low‐volume changes upon cycling, indicative of long‐life operation.

#### 4.2.1 (PO_4_)(P_2_O_7_)‐based Materials

**Figure 13:**
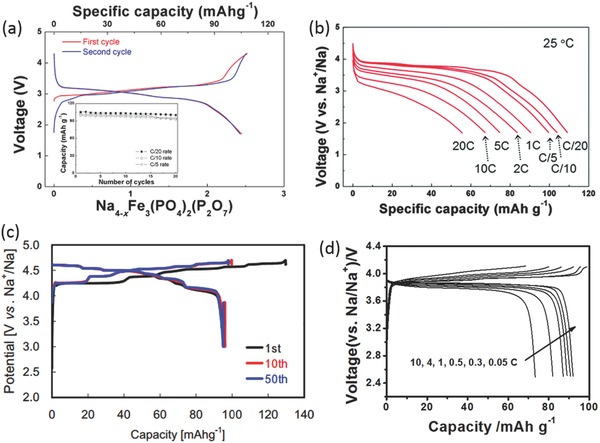
(a) Galvanostatic charge/discharge profiles of Na_4_Fe_3_(PO_4_)_2_(P_2_O_7_) in a Na‐ion cell at the C/20 rate; (b) The discharge profiles of Na_4_Mn_3_(PO_4_)_2_(P_2_O_7_) at various rates of C/20 to 20 C at 25 °C; (c) Galvanostatic charge–discharge curves at 1st, 10th and 50th cycles of Na_4_Co_3_(PO_4_)_2_P_2_O_7_; (d) The charge/discharge curves of the Na_7_V_4_(P_2_O_7_)_4_(PO_4_) nanorods at different current density. (a) Reproduced with permission.^230^ Copyright 2013, American Chemical Society. (b) Reproduced with permission.^231^ Copyright 2015, Royal Society of Chemistry. (c) Reproduced with permission.^232^ Copyright 2013, Elsevier. (d) Reproduced with permission.^236^ Copyright 2014, American Chemical Society.

Na_4_M_3_(PO_4_)_2_(P_2_O_7_) (M = Fe, Mn, Co, Ni) materials have been structurally characterized at early years and tested as electrochemically active materials for SIBs recently.^226^, ^227^, ^228^ The materials show double chains built up from PO_4_ tetrahedron and MO_6_ octahedra sharing corners with interlayer linkages via P–O–P bridges of the pyrophosphate groups in such a way that large tunnels extending along the [010] and [001] directions occur between two neighboring sheets.^226^ Kang's group first studied the Li and Na storage performance of Na_4_Fe_3_(PO_4_)_2_(P_2_O_7_) through a combined first principles calculations and experiments and reported a reversible capacity of 129 mAh g^–1^ and average potential of 3 V for the Na‐ion cell (**Figure** **13**a).^229^ The Na storage mechanism of Na_4_Fe_3_(PO_4_)_2_(P_2_O_7_) was testified to be a one‐phase reaction accompanying an exceptionally small volumetric change of less than 4%.^230^ They also extended the study to a Mn‐based material, Na_4_Mn_3_(PO_4_)_2_(P_2_O_7_), as cathodes for SIBs. The Na_4_Mn_3_(PO_4_)_2_(P_2_O_7_) material exhibited a largest Mn^2+^/Mn^3+^ redox potential of 3.84 V yet reported for a manganese‐based cathode for SIBs, and a reversible capacity of 109 mAh g^–1^. First‐principles calculations and experimental study showed that three‐dimensional Na diffusion pathways with low activation energy ensured the high rate capability of the material (20 C rate) (Figure 13b), and it was worth to be noted that the structural distortion induced by Jahn‐Teller distortion could open up sodium diffusion channels thus to increase the sodium ion mobility.^231^ Na_4_Co_3_(PO_4_)_2_P_2_O_7_ material has been employed as cathode for SIBs by Nose et al, which showed multi redox couples in the high potential region between 4.1 and 4.7 V with reversible capacity of 97 mAh g^–1^ (Figure 13c).^232^ Density functional theory calculations indicated that the removal of Na down to NaCo_3_(PO_4_)_2_P_2_O_7_ was found to be accompanied by oxidation of Co^2+^ to Co^3+^, and further removal of Na to give Co_3_(PO_4_)_2_P_2_O_7_ requires oxidation of oxygen 2*p* orbitals in the P_2_O_7_ polyhedra instead of Co^3+^ being oxidized to Co^4+^.^233^ They also reported a Na_4_Co_2.4_Mn_0.3_Ni_0.3_(PO_4_)_2_P_2_O_7_ material with two redox couples around 4.2 and 4.6 V with a capacity of 110 mAh g^–1^.^234^

Lim et al. have reported a V‐based mixed polyanion material, Na_7_V_4_(P_2_O_7_)_4_(PO_4_) with a tetragonal structure (a space group of *P* $\bar{4}$2_1_ *c*). The Na_7_V_4_(P_2_O_7_)_4_(PO_4_) holds exceptional electrochemical properties represented by well‐defined high voltage profiles at 3.88 V (Figure 13d) and substantial capacity retention over 1000 cycles.^235^ The material showed a V^3+^/V^4+^ redox reaction with Na_5_V^3.5+^_4_(P_2_O_7_)_4_(PO_4_) as intermediate phase, resulting in two plateaus in charge/discharge curves.^236^ Through elaborate structure design and conductive carbon coating, the Na_7_V_4_(P_2_O_7_)_4_(PO_4_) material showed a high rate capability of 30 C with excellent cycling stability of 94% capacity retention over 800 cycles.^237^

#### 4.2.2 (PO_4_)(CO_3_)‐based Materials

**Figure 14:**
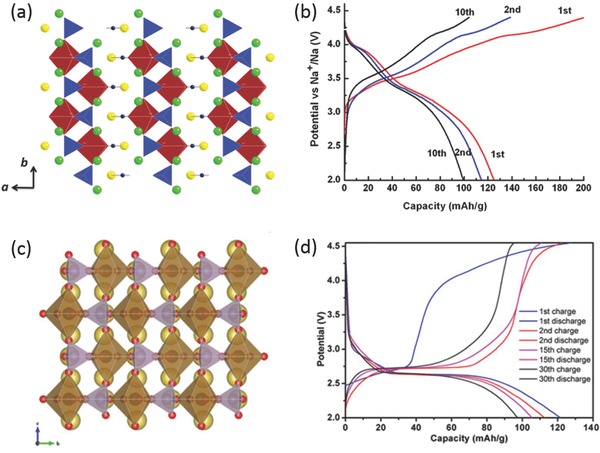
(a) The structure of Na_3_MnPO_4_CO_3_ viewed along [001]; (b) The voltage curves of sidorenkite Na_3_MnPO_4_CO_3_ at the first, second, and 10th cycles with a C/100 rate; (c) Geometrical model of the Na_3_FePO_4_CO_3_ along the a axis; (d) Galvanostatic charge/discharge profiles of Na_3_FePO_4_CO_3_ nanoplates at 10 mA/g for different cycles. (a,b) Reproduced with permission.^238^ Copyright 2013, American Chemical Society. (c,d) Reproduced with permission.^241^ Copyright 2014, Nature Publishing Group.

PO_4_ and CO_3_ can also be combined together to construct novel structure of carbonophosphates. Ceder's group reported a Na_3_MnPO_4_CO_3_ material with a reversible capacity of 125 mAh g^−1^ and average potential of 3.3 V (**Figure** **14**b). In situ X‐ray diffraction measurement suggested that the sidorenkite Na_3_MnPO_4_CO_3_ underwent a solid solution type reversible topotactic structural evolution upon electrochemical cycling.^238^ After that, Na_3_MnPO_4_CO_3_ electrodes with improved reversible capacity were reported.^239^, ^240^ Hassanzadeh et al. revealed a Na_3_MnCO_3_PO_4_/rGO hybrid with a high reversible capacity of 156 mAh g^–1^.^240^ However, the Na_3_MnCO_3_PO_4_ electrode exhibited high charge/discharge polarization with fast capacity degradation. Huang et al. reported another carbonophosphate, Na_3_FeCO_3_PO_4_, with a reversible capacity of 120 mAh g^–1^ and average working potential of 2.6 V (Figure 14d). In situ experiments indicated that both Fe^2+^/Fe^3+^ and Fe^3+^/Fe^4+^ redox couples were electrochemically active.^241^

## 5 New Perspectives of Phosphate Framework Materials

### 5.1 Aqueous Sodium Ion Batteries

Batteries using aqueous electrolyte can reduce the cost and be safer. Due to the very stable structure of phosphate materials, many of the materials have been shown excellent electrochemical performance in aqueous batteries.

Considering the proper redox potential and highly stability, NaTi_2_(PO_4_)_3_ has been widely accepted as an ideal candidate as anodes for aqueous SIBs.^242^ It exhibited a moderately high reversible capacity of 124 mAh g^–1^ and the plateau voltage was –0.8 V vs. Ag/AgCl reference electrode. By conductive carbon decoration, the rate capability and cycle stability of the NaTi_2_(PO_4_)_3_ electrodes in aqueous SIBs can be largely enhanced.^243^, ^244^, ^245^, ^246^, ^247^ For example, Li et al. reported a graphene decorated NaTi_2_(PO_4_)_3_, which exhibited high rate capability of 20 C and a long cycle life with a capacity retention of 71% over 2000 cycles.^245^ A number of aqueous SIBs have also been constructed by using NaTi_2_(PO_4_)_3_ anodes, such as the NaTi_2_(PO_4_)_3_//Na_2_NiFe(CN)_6_,^248^ NaTi_2_(PO_4_)_3_//Na_0.44_MnO_2_,^249^, ^250^, ^251^, ^252^ NaTi_2_(PO_4_)_3_//Na_3_V_2_(PO_4_)_3_,^253^ NaTi_2_(PO_4_)_3_//Na_2_FeP_2_O_7_,^254^ NaTi_2_(PO_4_)_3_//NaFePO_4_,^255^ and NaTi_2_(PO_4_)_3_//Na_3_V_2_O_2x_(PO_4_)_2_F_3‐2x_ couples.^256^

Other phosphate materials have been investigated as electrode materials for aqueous SIBs, for example, the Na_2_FeP_2_O_7_,^257^ Na_3_V_2_(PO_4_)_3_,^258^, ^259^ Na_2_VTi(PO_4_)_3_,^260^ Na_7_V_4_(P_2_O_7_)_4_(PO_4_),^261^ Na_3_V_2_O_2_(PO_4_)_2_F.^262^ However, due to the partial dissolution of electrode materials and inappropriate redox potential (close to the potential of hydrogen or oxygen evolution), these materials cannot be widely accepted as electrodes for large scale application. More efforts should be made to explore novel materials or elaborated structure design to gain electrodes with high performance in aqueous SIBs.

### 5.2 Ab Initio Computations

Novel materials are the key to the development of electrodes for energy storage system, however, conventional discovery of compounds with new structure and electrochemical activity requires large amount of repetitive experiments, considering the uncertain synthetic conditions and parameters. Ab initio computations in the density functional theory could be used to provide insight into the fundamental properties of electrode materials of lithium ion batteries.^263^, ^264^ Ab initio computations are accurate enough to understand and even predict electrode properties (eg., voltage, lithium diffusion, stability, and safety). Using a computational high‐throughput approach of computing properties on thousands of materials, the high scalability of computing can offer the possibility to discover new electrode materials.^265^, ^266^

Phosphate materials have been evaluated as electrode materials for lithium ion batteries by using high‐throughput ab initio computations. The limits and opportunities for the phosphate chemistry in terms of voltage, capacity (gravimetric and volumetric), specific energy, energy density, and safety were analyzed and discussed.^265^, ^267^ Using the same model and database, phosphate materials, even other kind of materials, can be evaluated and analyzed, which can help the experimental process of exploring new electrode materials for SIBs.^268^, ^269^, ^270^

## 6 Conclusions and Outlook

Sodium ion batteries have attracted increasing attention due to the wide availability and low cost of sodium resources. Exploring electrodes with higher structural and thermal stability is the key to promote electrochemical performance (long‐term cyclability and rate capability) of the SIBs for large scale energy storage application. From the structural point of view, phosphate materials possess the robust framework and exhibit low structure expansion or distortion, the potentially high operating voltages due to the inductive effect of phosphate groups or fluorophosphates groups, and high rate capability and long cycling life for high‐performance SIBs. The phosphates with proper reduction reaction potential should be the promising candidate for future energy storage application.

**Figure 15:**
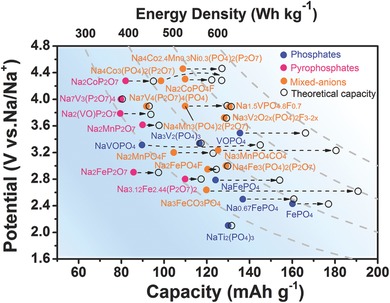
Phosphate framework materials and corresponding electrochemical data in the SIB technologies.

Phosphate framework materials are full of variety, exhibiting versatile and adjustable structure and electrochemical performance. Such as the phosphates, pyrophosphates, mixed‐anions, and optional redox centers (Fe, Mn, Co, V, Ni, et al.). The voltages and capacities (practical and theoretical) of representative phosphate framework materials for SIBs are summarized in **Figure** **15**. It's worth noting that some of the materials have shown high energy density (vs. metal Na anode) around 500 Wh kg^–1^, namely the Na_1.5_VPO_4.8_F_0.7_ and Na_3_V_2_O_2x_(PO_4_)_2_F_3–2x_, which exceed that of Li/LiMn_2_O_4_ (429 Wh kg^–1^) and are very close to those of Li/LiFePO4 (510 Wh kg^–1^) and Li/LiCoO2 (530 Wh kg^–1^), showing potential application for high energy density SIBs. Additionally, it can be seen that the exhibited capacities of some materials have been very close to their theoretical values, for example the NaTi_2_(PO_4_)_3_, Na_3_V_2_(PO_4_)_3_, and Na_3_V_2_O_2x_(PO_4_)_2_F_3–2x_. Therefore, most of the materials deliver capacities far lower than the theoretical capacities, for example the olivine NaFePO_4_, NaVOPO_4_, Na_3_MnPO_4_CO_3_, Na_4_Mn_3_(PO_4_)_2_(P_2_O_7_) and so on. Particularly, some systems such as Na/Na_3_MnPO_4_CO_3_ and Na/Na_4_Mn_3_(PO_4_)_2_(P_2_O_7_) might reach the theoretical energy density of 600 Wh kg^–1^. Thus, there will be a lot of work to do to improve the capacities of these materials in the future.

On the other hand, due to the intrinsic poor electron conductivity of phosphate materials, it is important and effective to improve the electrochemical performance of phosphate materials by carbon decoration. Enhancing the potential and capacity of electrode materials is also the important strategy to obtain SIBs with a high energy density. In addition, full cells based on phosphate materials should also be paid attention to accelerate the application of SIBs.

## References

[1] Q. Zhang , E. Uchaker , S. L. Candelaria , G. Cao , Chem. Soc. Rev. 2013, 42, 3127. 23455759 10.1039/c3cs00009e

[2] Z. Yang , J. Zhang , M. C. W. Kintner‐Meyer , X. Lu , D. Choi , J. P. Lemmon , J. Liu , Chem. Rev. 2011, 111, 3577. 21375330 10.1021/cr100290v

[3] H. Kim , H. Kim , Z. Ding , M. H. Lee , K. Lim , G. Yoon , K. Kang , Adv. Energy Mater. 2016, 6, 600943.

[4] C. Fang , Y. Huang , W. Zhang , J. Han , Z. Deng , Y. Cao , H. Yang , Adv. Energy Mater. 2016, 6, 501727.

[5] H. Pan , Y.‐S. Hu , L. Chen , Energy Environ. Sci. 2013, 6, 2338.

[6] Y. Cao , L. Xiao , W. Wang , D. Choi , Z. Nie , J. Yu , L. V. Saraf , Z. Yang , J. Liu , Adv. Mater. 2011, 23, 3155. 21638349 10.1002/adma.201100904

[7] D. Yuan , X. Liang , L. Wu , Y. Cao , X. Ai , J. Feng , H. Yang , Adv. Mater. 2014, 26, 6301. 25070474 10.1002/adma.201401946

[8] Y. Wang , R. Xiao , Y.‐S. Hu , M. Avdeev , L. Chen , Nat. Commun. 2015, 6, 6954. 25907679 10.1038/ncomms7954 PMC4421853

[9] S. Guo , P. Liu , H. Yu , Y. Zhu , M. Chen , M. Ishida , H. Zhou , Angew. Chem. 2015, 127, 5992. 10.1002/anie.201411788 25833039

[10] Y. Fang , L. Xiao , J. Qian , X. Ai , H. Yang , Y. Cao , Nano Lett. 2014, 14, 3539. 24857545 10.1021/nl501152f

[11] Y. Fang , L. Xiao , X. Ai , Y. Cao , H. Yang , Adv. Mater. 2015, 27, 5895. 26305519 10.1002/adma.201502018

[12] X. Rui , W. Sun , C. Wu , Y. Yu , Q. Yan , Adv. Mater. 2015, 27, 6670. 26417996 10.1002/adma.201502864

[13] J. Qian , M. Zhou , Y. Cao , X. Ai , H. Yang , Adv. Energy Mater. 2012, 2, 410.

[14] Y. You , X.‐L. Wu , Y.‐X. Yin , Y.‐G. Guo , Energy Environ. Sci. 2014, 7, 1643.

[15] L. Wang , Y. Lu , J. Liu , M. Xu , J. Cheng , D. Zhang , J. B. Goodenough , Angew. Chem. Int. Ed. 2013, 52, 1964. 10.1002/anie.201206854 23319239

[16] L. Xiao , Y. Cao , W. A. Henderson , M. L. Sushko , Y. Shao , J. Xiao , W. Wang , M. H. Engelhard , Z. Nie , J. Liu , Nano Energy 2016, 19, 279.

[17] S. Komaba , W. Murata , T. Ishikawa , N. Yabuuchi , T. Ozeki , T. Nakayama , A. Ogata , K. Gotoh , K. Fujiwara , Adv. Funct. Mater. 2011, 21, 3859.

[18] W. Luo , J. Schardt , C. Bommier , B. Wang , J. Razink , J. Simonsen , X. Ji , J. Mater. Chem. A 2013, 1, 10662.

[19] Y. Cao , L. Xiao , M. L. Sushko , W. Wang , B. Schwenzer , J. Xiao , Z. Nie , L. V. Saraf , Z. Yang , J. Liu , Nano Lett. 2012, 12, 3783. 22686335 10.1021/nl3016957

[20] L. Wu , X. Hu , J. Qian , F. Pei , F. Wu , R. Mao , X. Ai , H. Yang , Y. Cao , Energy Environ. Sci. 2014, 7, 323.

[21] J. Qian , X. Wu , Y. Cao , X. Ai , H. Yang , Angew. Chem. 2013, 125, 4731. 10.1002/anie.201209689 23512686

[22] L. Xiao , Y. Cao , J. Xiao , W. Wang , L. Kovarik , Z. Nie , J. Liu , Chem. Commun. 2012, 48, 3321. 10.1039/c2cc17129e 22361490

[23] R. Zhao , L. Zhu , Y. Cao , X. Ai , H. X. Yang , Electrochem. Commun. 2012, 21, 36.

[24] W. Luo , M. Allen , V. Raju , X. Ji , Adv. Energy Mater. 2014, 4, 4000554.

[25] S. Wang , L. Wang , Z. Zhu , Z. Hu , Q. Zhao , J. Chen , Angew. Chem. Int. Ed. 2014, 53, 5892. 10.1002/anie.201400032 24677513

[26] J. W. Wang , X. H. Liu , S. X. Mao , J. Y. Huang , Nano Lett. 2012, 12, 5897. 23092238 10.1021/nl303305c

[27] L. Wang , J. Song , R. Qiao , L. A. Wray , M. A. Hossain , Y.‐D. Chuang , W. Yang , Y. Lu , D. Evans , J.‐J. Lee , S. Vail , X. Zhao , M. Nishijima , S. Kakimoto , J. B. Goodenough , J. Am. Chem. Soc. 2015, 137, 2548. 25615887 10.1021/ja510347s

[28] R. Roy , D. K. Agrawal , H. A. McKinstry , Ann. Rev. Mater. Sci. 1989, 19, 59.

[29] C. Masquelier , L. Croguennec , Chem. Rev. 2013, 113, 6552. 23742145 10.1021/cr3001862

[30] K. Zaghib , J. Trottier , P. Hovington , F. Brochu , A. Guerfi , A. Mauger , C. M. Julien , J. Power Sources 2011, 196, 9612.

[31] P. Moreau , D. Guyomard , J. Gaubicher , F. Boucher , Chem. Mater. 2010, 22, 4126.

[32] P. P. Prosini , C. Cento , A. Masci , M. Carewska , Solid State Ionics 2014, 263, 1.

[33] N. Le Poul , E. Baudrin , M. Morcrette , S. Gwizdala , C. Masquelier , J. M. Tarascon , Solid State Ionics 2003, 159, 149.

[34] F. Boucher , J. Gaubicher , M. Cuisinier , D. Guyomard , P. Moreau , J. Am. Chem. Soc. 2014, 136, 9144. 24877619 10.1021/ja503622y

[35] J. Gaubicher , F. Boucher , P. Moreau , M. Cuisinier , P. Soudan , E. Elkaim , D. Guyomard , Electrochem. Commun. 2014, 38, 104.

[36] S.‐M. Oh , S.‐T. Myung , J. Hassoun , B. Scrosati , Y.‐K. Sun , Electrochem. Commun. 2012, 22, 149.

[37] M. Casas‐Cabanas , V. V. Roddatis , D. Saurel , P. Kubiak , J. Carretero‐Gonzalez , V. Palomares , P. Serras , T. Rojo , J. Mater. Chem. 2012, 22, 17421.

[38] M. Galceran , V. Roddatis , F. J. Zúñiga , J. M. Pérez‐Mato , B. Acebedo , R. Arenal , I. Peral , T. Rojo , M. Casas‐Cabanas , Chem. Mater. 2014, 26, 3289.

[39] M. Galceran , D. Saurel , B. Acebedo , V. V. Roddatis , E. Martin , T. Rojo , M. Casas‐Cabanas , Phys. Chem. Chem. Phys. 2014, 16, 8837. 24676033 10.1039/c4cp01089b

[40] A. Saracibar , J. Carrasco , D. Saurel , M. Galceran , B. Acebedo , H. Anne , M. Lepoitevin , T. Rojo , M. Casas Cabanas , Phys. Chem. Chem. Phys. 2016, 18, 13045. 27110665 10.1039/c6cp00762g

[41] J. Lu , S. C. Chung , S.‐i. Nishimura , A. Yamada , Chem. Mater. 2013, 25, 4557.

[42] S. J. Tracy , L. Mauger , H. L. Smith , H. J. Tan , J. E. Herriman , Y. Xiao , B. Fultz , Chem. Mater. 2016, 28, 3051.

[43] J. Sugiyama , H. Nozaki , M. Harada , Y. Higuchi , J. H. Brewer , E. J. Ansaldo , G. Kobayashi , R. Kanno , Phys. Rev.B 2014, 90, 014426.

[44] Y. Fang , Q. Liu , L. Xiao , X. Ai , H. Yang , Y. Cao , ACS Appl. Mater. Interfaces 2015, 7, 17977. 26207862 10.1021/acsami.5b04691

[45] W. Tang , X. Song , Y. Du , C. Peng , M. Lin , S. Xi , B. Tian , J. Zheng , Y. Wu , F. Pan , K. P. Loh , J. Mater. Chem. A 2016, 4, 4882.

[46] G. Ali , J.‐H. Lee , D. Susanto , S.‐W. Choi , B. W. Cho , K.‐W. Nam , K. Y. Chung , ACS Appl. Mater. Interfaces 2016, 8, 15422. 27248477 10.1021/acsami.6b04014

[47] N. Wongittharom , T.‐C. Lee , C.‐H. Wang , Y.‐C. Wang , J.‐K. Chang , J. Mater. Chem. A 2014, 2, 5655.

[48] N. Wongittharom , C.‐H. Wang , Y.‐C. Wang , C.‐H. Yang , J.‐K. Chang , ACS Appl. Mater. Interfaces 2014, 6, 17564. 25295391 10.1021/am5033605

[49] Y. Zhu , Y. Xu , Y. Liu , C. Luo , C. Wang , Nanoscale 2013, 5, 780. 23235803 10.1039/c2nr32758a

[50] A. Whiteside , C. A. J. Fisher , S. C. Parker , M. Saiful Islam , Phys. Chem. Chem. Phys. 2014, 16, 21788. 25200320 10.1039/c4cp02356k

[51] M. Nakayama , S. Yamada , R. Jalem , T. Kasuga , Solid State Ionics 2016, 286, 40.

[52] M. Dixit , H. Engel , R. Eitan , D. Aurbach , M. D. Levi , M. Kosa , D. T. Major , J. Phys. Chem. C 2015, 119, 15801.

[53] T. Boyadzhieva , V. Koleva , E. Zhecheva , D. Nihtianova , L. Mihaylov , R. Stoyanova , RSC Adv. 2015, 5, 87694.

[54] K. T. Lee , T. N. Ramesh , F. Nan , G. Botton , L. F. Nazar , Chem. Mater. 2011, 23, 3593.

[55] M. Avdeev , Z. Mohamed , C. D. Ling , J. Lu , M. Tamaru , A. Yamada , P. Barpanda , Inorg. Chem. 2013, 52, 8685. 23844797 10.1021/ic400870x

[56] H. H. Kim , I. H. Yu , H. S. Kim , H.‐J. Koo , M.‐H. Whangbo , Inorg. Chem. 2015, 54, 4966. 25928193 10.1021/acs.inorgchem.5b00577

[57] J. Kim , D.‐H. Seo , H. Kim , I. Park , J.‐K. Yoo , S.‐K. Jung , Y.‐U. Park , W. A. Goddard III , K. Kang , Energy Environ. Sci. 2015, 8, 540.

[58] W. Huang , J. Zhou , B. Li , L. An , P. Cui , W. Xia , L. Song , D. Xia , W. Chu , Z. Wu , Small 2015, 11, 2170. 25641786 10.1002/smll.201402246

[59] K. Trad , D. Carlier , L. Croguennec , A. Wattiaux , M. Ben Amara , C. Delmas , Chem. Mater. 2010, 22, 5554. 10.1021/ic101130m 20949928

[60] K. Saravanan , C. W. Mason , A. Rudola , K. H. Wong , P. Balaya , Adv. Energy Mater. 2013, 3, 444.

[61] N. Anantharamulu , K. Koteswara Rao , G. Rambabu , B. Vijaya Kumar , V. Radha , M. Vithal , J. Mater. Sci. 2011, 46, 2821.

[62] P. Knauth , Solid State Ionics 2009, 180, 911.

[63] M. Cretin , P. Fabry , J. Eur. Ceram. Soc. 1999, 19, 2931.

[64] J. B. Goodenough , H. Y. P. Hong , J. A. Kafalas , Mater. Res. Bull. 1976, 11, 203.

[65] L. Vijayan , G. Govindaraj , NASICON Materials: structure and electrical properties, INTECH Open Access Publisher, 2012.

[66] H.‐P. Hong , Mater. Res. Bull. 1976, 11, 173.

[67] D. B. Porter , R. Olazcuaga , C. Delmas , F. Cherkaoui , R. Brochu , G. Leflem , REVUE DE CHIMIE MINERALE 1980, 17, 458.

[68] W. Song , X. Ji , Z. Wu , Y. Zhu , Y. Yang , J. Chen , M. Jing , F. Li , C. E. Banks , J. Mater. Chem. A 2014, 2, 5358.

[69] S. Y. Lim , H. Kim , R. A. Shakoor , Y. Jung , J. W. Choi , J. Electrochem. Soc. 2012, 159, A1393.

[70] J.‐N. Chotard , G. Rousse , R. David , O. Mentré , M. Courty , C. Masquelier , Chem. Mater. 2015, 27, 5982.

[71] Y. Uebou , T. Kiyabu , The Reports of Institute of Adv. Mater. Study 2002, 16, 1.

[72] L. S. Plashnitsa , E. Kobayashi , Y. Noguchi , S. Okada , J.‐i. Yamaki , J. Electrochem. Soc. 2010, 157, A536.

[73] Z. Jian , L. Zhao , H. Pan , Y.‐S. Hu , H. Li , W. Chen , L. Chen , Electrochem. Commun. 2012, 14, 86.

[74] Z. Jian , W. Han , X. Lu , H. Yang , Y.‐S. Hu , J. Zhou , Z. Zhou , J. Li , W. Chen , D. Chen , L. Chen , Adv. Energy Mater. 2013, 3, 156.

[75] Z. Jian , C. Yuan , W. Han , X. Lu , L. Gu , X. Xi , Y.‐S. Hu , H. Li , W. Chen , D. Chen , Y. Ikuhara , L. Chen , Adv. Funct. Mater. 2014, 24, 4265.

[76] W. Song , X. Cao , Z. Wu , J. Chen , K. Huangfu , X. Wang , Y. Huang , X. Ji , Phys. Chem. Chem. Phys. 2014, 16, 17681. 25028981 10.1039/c4cp01821d

[77] K. M. Bui , V. A. Dinh , S. Okada , T. Ohno , Phys. Chem. Chem. Phys. 2015, 17, 30433. 26509737 10.1039/c5cp05323d

[78] S. Kajiyama , K. Kai , M. Okubo , A. Yamada , Electrochem. 2016, 84, 234.

[79] M. Pivko , I. Arcon , M. Bele , R. Dominko , M. Gaberscek , J. Power Sources 2012, 216, 145.

[80] F. A. Nizamov , P. N. Togulev , D. R. Abdullin , S. M. Khantimerov , P. Balaya , N. M. Suleimanov , Phys. Solid State 2016, 58, 475.

[81] S.‐J. Lim , D.‐W. Han , D.‐H. Nam , K.‐S. Hong , J.‐Y. Eom , W.‐H. Ryu , H.‐S. Kwon , J. Mater. Chem. A 2014, 2, 19623.

[82] H. Li , X. Yu , Y. Bai , F. Wu , C. Wu , L.‐Y. Liu , X.‐Q. Yang , J. Mater. Chem. A 2015, 3, 9578.

[83] G. Xu , G. Sun , Ceram. Int. 2016, 42, 14774.

[84] M. J. Aragón , P. Lavela , G. F. Ortiz , J. L. Tirado , J. Electrochem. Soc. 2015, 162, A3077.

[85] M. J. Aragón , P. Lavela , G. F. Ortiz , J. L. Tirado , ChemElectroChem 2015, 2, 995.

[86] R. Klee , P. Lavela , M. J. Aragón , R. Alcántara , J. L. Tirado , J. Power Sources 2016, 313, 73.

[87] M. J. Aragón , P. Lavela , R. Alcántara , J. L. Tirado , Electrochim. Acta 2015, 180, 824.

[88] C. W. Mason , I. Gocheva , H. E. Hoster , D. Y. W. Yu , ECS Transactions 2014, 58, 41.

[89] B. M. de Boisse , J. Ming , S.‐i. Nishimura , A. Yamada , J. Electrochem. Soc. 2016, 163, A1469.

[90] S. Li , Y. Dong , L. Xu , X. Xu , L. He , L. Mai , Adv. Mater. 2014, 26, 3545. 24633680 10.1002/adma.201305522

[91] T.‐F. Hung , W.‐J. Cheng , W.‐S. Chang , C.‐C. Yang , C.‐C. Shen , Y.‐L. Kuo , Chem. Eur. J. 2016, 22, 10620. 27346677 10.1002/chem.201602066

[92] W. Shen , C. Wang , H. Liu , W. Yang , Chem. Eur. J. 2013, 19, 14712. 24014393 10.1002/chem.201300005

[93] J. Mao , C. Luo , T. Gao , X. Fan , C. Wang , J. Mater. Chem. A 2015, 3, 10378.

[94] Q. Zhang , W. Wang , Y. Wang , P. Feng , K. Wang , S. Cheng , K. Jiang , Nano Energy 2016, 20, 11.

[95] H. Wang , D. Jiang , Y. Zhang , G. Li , X. Lan , H. Zhong , Z. Zhang , Y. Jiang , Electrochim. Acta 2015, 155, 23.

[96] S. Tao , P. Cui , W. Huang , Z. Yu , X. Wang , S. Wei , D. Liu , L. Song , W. Chu , Carbon 2016, 96, 1028.

[97] L. Si , Z. Yuan , L. Hu , Y. Zhu , Y. Qian , J. Power Sources 2014, 272, 880.

[98] Z. Chu , C. Yue , Solid State Ionics 2016, 287, 36.

[99] W. Duan , Z. Zhu , H. Li , Z. Hu , K. Zhang , F. Cheng , J. Chen , J. Mater. Chem. A 2014, 2, 8668.

[100] X. Zhu , Y. Fang , X. Ai , H. Yang , Y. Cao , J. Alloys Compd 2015, 646, 170.

[101] G. Li , D. Jiang , H. Wang , X. Lan , H. Zhong , Y. Jiang , J. Power Sources 2014, 265, 325.

[102] R. Klee , M. J. Aragón , R. Alcántara , J. L. Tirado , P. Lavela , Eur. J. Inorg. Chem. 2016, 2016, 3212.

[103] P. Nie , Y. Zhu , L. Shen , G. Pang , G. Xu , S. Dong , H. Dou , X. Zhang , J. Mater. Chem. A 2014, 2, 18606.

[104] W. Shen , H. Li , C. Wang , Z. Li , Q. Xu , H. Liu , Y. Wang , J. Mater. Chem. A 2015, 3, 15190.

[105] J.‐Z. Guo , X.‐L. Wu , F. Wan , J. Wang , X.‐H. Zhang , R.‐S. Wang , Chem. Eur. J. 2015, 21, 17371. 26481446 10.1002/chem.201502583

[106] J. Liu , K. Tang , K. Song , P. A. van Aken , Y. Yu , J. Maier , Nanoscale 2014, 6, 5081. 24595960 10.1039/c3nr05329f

[107] S. Kajiyama , J. Kikkawa , J. Hoshino , M. Okubo , E. Hosono , Chem. Eur. J. 2014, 20, 12636. 25123497 10.1002/chem.201403126

[108] H. Li , Y. Bai , F. Wu , Y. Li , C. Wu , J. Power Sources 2015, 273, 784.

[109] Y. H. Jung , C. H. Lim , D. K. Kim , J. Mater. Chem. A 2013, 1, 11350.

[110] S. Tao , X. Wang , P. Cui , Y. Wang , Y. A. Haleem , S. Wei , W. Huang , L. Song , W. Chu , RSC Adv. 2016, 6, 43591.

[111] J. Fang , S. Wang , Z. Li , H. Chen , L. Xia , L. Ding , H. Wang , J. Mater. Chem. A 2016, 4, 1180.

[112] Y. Xu , Q. Wei , C. Xu , Q. Li , Q. An , P. Zhang , J. Sheng , L. Zhou , L. Mai , Adv. Energy Mater. 2016, 600389.

[113] C. Zhu , P. Kopold , P. A. van Aken , J. Maier , Y. Yu , Adv. Mater. 2016, 28, 2409. 26810919 10.1002/adma.201505943

[114] C. Zhu , K. Song , P. A. van Aken , J. Maier , Y. Yu , Nano Lett. 2014, 14, 2175. 24678829 10.1021/nl500548a

[115] W. Shen , H. Li , Z. Guo , C. Wang , Z. Li , Q. Xu , H. Liu , Y. Wang , Y. Xia , ACS Appl. Mater. Interfaces 2016, 8, 15341. 27257712 10.1021/acsami.6b03410

[116] Y. Jiang , Z. Yang , W. Li , L. Zeng , F. Pan , M. Wang , X. Wei , G. Hu , L. Gu , Y. Yu , Adv. Energy Mater. 2015, DOI: 10.1002/aenm.201402104.

[117] Z. Jian , Y. Sun , X. Ji , Chem. Commun. 2015, 51, 6381. 10.1039/c5cc00944h 25762199

[118] D. Wang , N. Chen , M. Li , C. Wang , H. Ehrenberg , X. Bie , Y. Wei , G. Chen , F. Du , J. Mater. Chem. A 2015, 3, 8636.

[119] Y. Dong , S. Qian , L. Shao , H. Yu , L. Yan , P. Li , X. Lin , N. Long , M. Shui , J. Shu , Ceram. Int. 2016, 42, 10943.

[120] Y. Zhang , H. Zhao , Y. Du , J. Mater. Chem. A 2016, 4, 7155.

[121] F. Lalère , J. B. Leriche , M. Courty , S. Boulineau , V. Viallet , C. Masquelier , V. Seznec , J. Power Sources 2014, 247, 975.

[122] L. O. Hagman , P. Kierkegaard , Acta Chem. Scand. 1968, 22, 1822.

[123] C. Delmas , F. Cherkaoui , A. Nadiri , P. Hagenmuller , Mater. Res. Bull. 1987, 22, 631.

[124] G. Pang , P. Nie , C. Yuan , L. Shen , X. Zhang , H. Li , C. Zhang , J. Mater. Chem. A 2014, 2, 20659.

[125] G. Pang , C. Yuan , P. Nie , J. Zhu , X. Zhang , H. Li , B. Ding , Appl. Mater. Today 2016, 4, 54.

[126] J. Yang , H. Wang , P. Hu , J. Qi , L. Guo , L. Wang , Small 2015, 11, 3744. 25919707 10.1002/smll.201500144

[127] L. Wang , B. Wang , G. Liu , T. Liu , T. Gao , D. Wang , RSC Adv. 2016, 6, 70277.

[128] Z. Huang , L. Liu , L. Yi , W. Xiao , M. Li , Q. Zhou , G. Guo , X. Chen , H. Shu , X. Yang , X. Wang , J. Power Sources 2016, 325, 474.

[129] G. Yang , H. Song , M. Wu , C. Wang , J. Mater. Chem. A 2015, 3, 18718.

[130] C. Wu , P. Kopold , Y.‐L. Ding , P. A. van Aken , J. Maier , Y. Yu , ACS Nano 2015, 9, 6610. 26053194 10.1021/acsnano.5b02787

[131] Y. Niu , M. Xu , Y. Zhang , J. Han , Y. Wang , C. M. Li , RSC Adv. 2016, 6, 45605.

[132] J. Song , S. Park , J. Gim , V. Mathew , S. Kim , J. Jo , S. Kim , J. Kim , J. Mater. Chem. A 2016, 4, 7815.

[133] H.‐K. Roh , H.‐K. Kim , M.‐S. Kim , D.‐H. Kim , K. Y. Chung , K. C. Roh , K.‐B. Kim , Nano Res. 2016, 9, 1844.

[134] Y. Jiang , J. Shi , M. Wang , L. Zeng , L. Gu , Y. Yu , ACS Appl. Mater. Interfaces 2016, 8, 689. 26653567 10.1021/acsami.5b09811

[135] Y. Fang , L. Xiao , J. Qian , Y. Cao , X. Ai , Y. Huang , H. Yang , Adv. Energy Mater. 2016, *in press*.

[136] P. Senguttuvan , G. Rousse , M. E. Arroyo y de Dompablo , H. Vezin , J. M. Tarascon , M. R. Palacín , J. Am. Chem. Soc. 2013, 135, 3897. 23421416 10.1021/ja311044t

[137] D. Wang , Q. Liu , C. Chen , M. Li , X. Meng , X. Bie , Y. Wei , Y. Huang , F. Du , C. Wang , G. Chen , ACS Appl. Mater. Interfaces 2016, 8, 2238. 26720111 10.1021/acsami.5b11003

[138] Y. Niu , M. Xu , C. Guo , C. M. Li , J. Coll. Interface Sci. 2016, 474, 88. 10.1016/j.jcis.2016.04.021 27108073

[139] M. J. Aragón , C. Vidal‐Abarca , P. Lavela , J. L. Tirado , J. Power Sources 2014, 252, 208.

[140] S. Difi , I. Saadoune , M. T. Sougrati , R. Hakkou , K. Edstrom , P.‐E. Lippens , Hyperfine Interac. 2016, 237, 1.

[141] S. Difi , I. Saadoune , M. T. Sougrati , R. Hakkou , K. Edstrom , P.‐E. Lippens , J. Phys. Chem. C 2015, 119, 25220.

[142] S. Alexander , Phys. Reports 1998, 296, 65.

[143] L. R. Hilden , K. R. Morris , J. Pharm. Sci. 2004, 93, 3. 14648630 10.1002/jps.10489

[144] T. Shiratsuchi , S. Okada , J. Yamaki , T. Nishida , J. Power Sources 2006, 159, 268.

[145] V. Mathew , S. Kim , J. Kang , J. Gim , J. Song , J. P. Baboo , W. Park , D. Ahn , J. Han , L. Gu , Y. Wang , Y.‐S. Hu , Y.‐K. Sun , J. Kim , NPG Asia Mater. 2014, 6, e138.

[146] J. Zhao , Z. Jian , J. Ma , F. Wang , Y.‐S. Hu , W. Chen , L. Chen , H. Liu , S. Dai , ChemSusChem 2012, 5, 1495. 22692812 10.1002/cssc.201100844

[147] Y. Liu , Y. Xu , X. Han , C. Pellegrinelli , Y. Zhu , H. Zhu , J. Wan , A. C. Chung , O. Vaaland , C. Wang , L. Hu , Nano Lett. 2012, 12, 5664. 23072358 10.1021/nl302819f

[148] S. Xu , S. Zhang , J. Zhang , T. Tan , Y. Liu , J. Mater. Chem. A 2014, 2, 7221.

[149] Y. Liu , S. Xu , S. Zhang , J. Zhang , J. Fan , Y. Zhou , J. Mater. Chem. A 2015, 3, 5501.

[150] Y. Liu , Y. Zhou , S. Zhang , J. Zhang , P. Ren , C. Qian , J. Solid State Electrochem. 2016, 20, 479.

[151] G. Yang , B. Ding , J. Wang , P. Nie , H. Dou , X. Zhang , Nanoscale 2016, 8, 8495. 27064740 10.1039/c6nr00409a

[152] W. Wang , S. Wang , H. Jiao , P. Zhan , S. Jiao , Phys. Chem. Chem. Phys. 2015, 17, 4551. 25582353 10.1039/c4cp05764c

[153] Y. Liu , Y. Zhou , J. Zhang , S. Zhang , P. Ren , J. Power Sources 2016, 314, 1.

[154] Y. Liu , Y. Zhou , J. Zhang , S. Zhang , S. Xu , Phys. Chem. Chem. Phys. 2015, 17, 22144. 26256115 10.1039/c5cp02059j

[155] T. Liu , Y. Duan , G. Zhang , M. Li , Y. Feng , J. Hu , J. Zheng , J. Chen , F. Pan , J. Mater. Chem. A 2016, 4, 4479.

[156] C. Li , X. Miao , W. Chu , P. Wu , D. G. Tong , J. Mater. Chem. A 2015, 3, 8265.

[157] P. Barpanda , S.‐i. Nishimura , A. Yamada , Adv. Energy Mater. 2012, 2, 841.

[158] P. Barpanda , T. Ye , S.‐i. Nishimura , S.‐C. Chung , Y. Yamada , M. Okubo , H. Zhou , A. Yamada , Electrochem. Commun. 2012, 24, 116.

[159] T. Honma , T. Togashi , N. Ito , T. Komatsu , J. Ceram. Soc. Japan 2012, 120, 344.

[160] P. Barpanda , G. Liu , C. D. Ling , M. Tamaru , M. Avdeev , S.‐C. Chung , Y. Yamada , A. Yamada , Chem. Mater. 2013, 25, 3480.

[161] J. M. Clark , P. Barpanda , A. Yamada , M. S. Islam , J. Mater. Chem. A 2014, 2, 11807.

[162] H. Kim , R. A. Shakoor , C. Park , S. Y. Lim , J.‐S. Kim , Y. N. Jo , W. Cho , K. Miyasaka , R. Kahraman , Y. Jung , J. W. Choi , Adv. Funct. Mater. 2013, 23, 1147.

[163] J. Ming , H. Ming , W. Yang , W.‐J. Kwak , J.‐B. Park , J. Zheng , Y.‐K. Sun , RSC Adv. 2015, 5, 8793.

[164] P. Barpanda , T. Ye , J. Lu , Y. Yamada , S.‐C. Chung , S. Nishimura , M. Okubo , H. Zhou , A. Yamada , ECS Transac. 2013, 50, 71.

[165] T. Honma , A. Sato , N. Ito , T. Togashi , K. Shinozaki , T. Komatsu , J. Non‐Crystalline Solids 2014, 404, 26.

[166] G. Longoni , J. E. Wang , Y. H. Jung , D. K. Kim , C. M. Mari , R. Ruffo , J. Power Sources 2016, 302, 61.

[167] C.‐Y. Chen , K. Matsumoto , T. Nohira , C. Ding , T. Yamamoto , R. Hagiwara , Electrochim. Acta 2014, 133, 583.

[168] C.‐Y. Chen , K. Matsumoto , T. Nohira , R. Hagiwara , Y. Orikasa , Y. Uchimoto , J. Power Sources 2014, 246, 783.

[169] R. A. Shakoor , C. S. Park , A. A. Raja , J. Shin , R. Kahraman , Phys. Chem. Chem. Phys. 2016, 18, 3929. 26765283 10.1039/c5cp06836c

[170] P. Barpanda , G. Liu , Z. Mohamed , C. D. Ling , A. Yamada , Solid State Ionics 2014, 268, Part B, 305.

[171] C.‐Y. Chen , K. Matsumoto , T. Nohira , R. Hagiwara , J. Electrochem. Soc. 2015, 162, A176.

[172] B. Lin , S. Zhang , C. Deng , J. Mater. Chem. A 2016, 4, 2550.

[173] Y. Niu , M. Xu , S.‐J. Bao , C. M. Li , Chem. Commun. 2015, 51, 13120. 10.1039/c5cc04422g 26191549

[174] Y. Niu , M. Xu , C. Cheng , S. Bao , J. Hou , S. Liu , F. Yi , H. He , C. M. Li , J. Mater. Chem. A 2015, 3, 17224.

[175] K.‐H. Ha , S. H. Woo , D. Mok , N.‐S. Choi , Y. Park , S. M. Oh , Y. Kim , J. Kim , J. Lee , L. F. Nazar , K. T. Lee , Adv. Energy Mater. 2013, 3, 770.

[176] C. S. Park , H. Kim , R. A. Shakoor , E. Yang , S. Y. Lim , R. Kahraman , Y. Jung , J. W. Choi , J. Am. Chem. Soc. 2013, 135, 2787. 23350583 10.1021/ja312044k

[177] P. Barpanda , T. Ye , M. Avdeev , S.‐C. Chung , A. Yamada , J. Mater. Chem. A 2013, 1, 4194.

[178] P. Barpanda , J. Lu , T. Ye , M. Kajiyama , S.‐C. Chung , N. Yabuuchi , S. Komaba , A. Yamada , RSC Adv. 2013, 3, 3857.

[179] P. Barpanda , M. Avdeev , C. D. Ling , J. Lu , A. Yamada , Inorg. Chem. 2013, 52, 395. 23244781 10.1021/ic302191d

[180] H. Kim , C. S. Park , J. W. Choi , Y. Jung , Angew. Chem. Int. Ed. 2016, 55, 6662. 10.1002/anie.201601022 27098851

[181] Y. Kee , N. Dimov , A. Staikov , P. Barpanda , Y.‐C. Lu , K. Minami , S. Okada , RSC Adv. 2015, 5, 64991.

[182] C. Deng , S. Zhang , B. Zhao , Energy Storage Mater. 2016, 4, 71.

[183] P. Barpanda , G. Liu , M. Avdeev , A. Yamada , ChemElectroChem 2014, 1, 1488.

[184] J. Kim , I. Park , H. Kim , K.‐Y. Park , Y.‐U. Park , K. Kang , Adv. Energy Mater. 2016, 6, 502147.

[185] N. R. Khasanova , O. A. Drozhzhin , D. A. Storozhilova , C. Delmas , E. V. Antipov , Chem. Mater. 2012, 24, 4271.

[186] B. L. Ellis , W. R. M. Makahnouk , Y. Makimura , K. Toghill , L. F. Nazar , Nat. Mater. 2007, 6, 749. 17828278 10.1038/nmat2007

[187] B. L. Ellis , W. R. M. Makahnouk , W. N. Rowan‐Weetaluktuk , D. H. Ryan , L. F. Nazar , Chem. Mater. 2010, 22, 1059.

[188] N. Recham , J.‐N. Chotard , L. Dupont , K. Djellab , M. Armand , J.‐M. Tarascon , J. Electrochem. Soc. 2009, 156, A993.

[189] A. Langrock , Y. Xu , Y. Liu , S. Ehrman , A. Manivannan , C. Wang , J. Power Sources 2013, 223, 62.

[190] Y. Kawabe , N. Yabuuchi , M. Kajiyama , N. Fukuhara , T. Inamasu , R. Okuyama , I. Nakai , S. Komaba , Electrochem. Commun. 2011, 13, 1225.

[191] J. Yan , X. Liu , B. Li , Electrochem. Commun. 2015, 56, 46.

[192] M. Law , V. Ramar , P. Balaya , RSC Adv. 2015, 5, 50155.

[193] R. Tripathi , S. M. Wood , M. S. Islam , L. F. Nazar , Energy Environ. Sci. 2013, 6, 2257.

[194] Y. Kawabe , N. Yabuuchi , M. Kajiyama , N. Fukuhara , T. Inamasu , R. Okuyama , I. Nakai , S. Komaba , Electrochem. 2012, 80, 80.

[195] S.‐W. Kim , D.‐H. Seo , H. Kim , K.‐Y. Park , K. Kang , Phys. Chem. Chem. Phys. 2012, 14, 3299. 22306916 10.1039/c2cp40082k

[196] X. Lin , X. Hou , X. Wu , S. Wang , M. Gao , Y. Yang , RSC Adv. 2014, 4, 40985.

[197] Y. Zheng , P. Zhang , S. Q. Wu , Y. H. Wen , Z. Z. Zhu , Y. Yang , J. Electrochem. Soc. 2013, 160, A927.

[198] K. Kubota , K. Yokoh , N. Yabuuchi , S. Komaba , Electrochem. 2014, 82, 909.

[199] H. Zou , S. Li , X. Wu , M. J. McDonald , Y. Yang , ECS Electrochem. Lett. 2015, 4, A53.

[200] J. Barker , M. Y. Saidi , J. L. Swoyer , Electrochem. Solid‐State Lett. 2003, 6, A1.

[201] Y. Lu , S. Zhang , Y. Li , L. Xue , G. Xu , X. Zhang , J. Power Sources 2014, 247, 770.

[202] Y.‐L. Ruan , K. Wang , S.‐D. Song , X. Han , B.‐W. Cheng , Electrochim. Acta 2015, 160, 330.

[203] H. Zhuo , X. Wang , A. Tang , Z. Liu , S. Gamboa , P. J. Sebastian , J. Power Sources 2006, 160, 698.

[204] Z.‐m. Liu , X.‐y. Wang , Y. Wang , A.‐p. Tang , S.‐y. Yang , L.‐f. He , Transac. Nonferrous Metals Soc. China 2008, 18, 346.

[205] P. Serras , V. Palomares , A. Goni , I. Gil de Muro , P. Kubiak , L. Lezama , T. Rojo , J. Mater. Chem. 2012, 22, 22301.

[206] F. Sauvage , E. Quarez , J. M. Tarascon , E. Baudrin , Solid State Sci. 2006, 8, 1215.

[207] J. M. Le Meins , M. P. Crosnier‐Lopez , A. Hemon‐Ribaud , G. Courbion , J. Solid State Chem. 1999, 148, 260.

[208] W. Song , X. Cao , Z. Wu , J. Chen , Y. Zhu , H. Hou , Q. Lan , X. Ji , Langmuir 2014, 30, 12438. 25212063 10.1021/la5025444

[209] J. Barker , R. K. B. Gover , P. Burns , A. J. Bryan , Electrochem. Solid‐State Lett. 2006, 9, A190.

[210] R. K. B. Gover , A. Bryan , P. Burns , J. Barker , Solid State Ionics 2006, 177, 1495.

[211] R. A. Shakoor , D.‐H. Seo , H. Kim , Y.‐U. Park , J. Kim , S.‐W. Kim , H. Gwon , S. Lee , K. Kang , J. Mater. Chem. 2012, 22, 20535.

[212] Y. Qi , L. Mu , J. Zhao , Y.‐S. Hu , H. Liu , S. Dai , J. Mater. Chem. A 2016, 4, 7178.

[213] Q. Liu , D. Wang , X. Yang , N. Chen , C. Wang , X. Bie , Y. Wei , G. Chen , F. Du , J. Mater. Chem. A 2015, 3, 21478.

[214] M. Bianchini , F. Fauth , N. Brisset , F. Weill , E. Suard , C. Masquelier , L. Croguennec , Chem. Mater. 2015, 27, 3009. 10.1107/S2052520615017199 26634725

[215] W. Song , X. Ji , Z. Wu , Y. Yang , Z. Zhou , F. Li , Q. Chen , C. E. Banks , J. Power Sources 2014, 256, 258.

[216] Z. Liu , Y.‐Y. Hu , M. T. Dunstan , H. Huo , X. Hao , H. Zou , G. Zhong , Y. Yang , C. P. Grey , Chem. Mater. 2014, 26, 2513.

[217] P. Serras , V. Palomares , A. Goñi , P. Kubiak , T. Rojo , J. Power Sources 2013, 241, 56.

[218] Y.‐U. Park , D.‐H. Seo , H. Kim , J. Kim , S. Lee , B. Kim , K. Kang , Adv. Funct. Mater. 2014, 24, 4603.

[219] Y. Qi , L. Mu , J. Zhao , Y.‐S. Hu , H. Liu , S. Dai , Angew. Chem. 2015, 127, 10049. 10.1002/anie.201503188 26179243

[220] P. Serras , V. Palomares , J. Alonso , N. Sharma , J. M. López del Amo , P. Kubiak , M. L. Fdez‐Gubieda , T. Rojo , Chem. Mater. 2013, 25, 4917.

[221] W. Massa , O. V. Yakubovich , O. V. Dimitrova , Solid State Sciences 2002, 4, 495.

[222] P. Serras , V. Palomares , P. Kubiak , L. Lezama , T. Rojo , Electrochem. Commun. 2013, 34, 344.

[223] N. Sharma , P. Serras , V. Palomares , H. E. A. Brand , J. Alonso , P. Kubiak , M. L. Fdez‐Gubieda , T. Rojo , Chem. Mater. 2014, 26, 3391.

[224] Y. U. Park , D. H. Seo , H. S. Kwon , B. Kim , J. Kim , H. Kim , I. Kim , H. I. Yoo , K. Kang , J. Am. Chem. Soc. 2013, 135, 13870. 23952799 10.1021/ja406016j

[225] M. Peng , B. Li , H. Yan , D. Zhang , X. Wang , D. Xia , G. Guo , Angew. Chem. Int. Ed. 2015, 54, 6452. 10.1002/anie.201411917 25864686

[226] F. Sanz , C. Parada , J. M. Rojo , C. Ruíz‐Valero , Chem. Mater. 2001, 13, 1334.

[227] F. Sanz , C. Parada , U. Amador , M. A. Monge , C. R. Valero , J. Solid State Chem. 1996, 123, 129.

[228] S. M. Wood , C. Eames , E. Kendrick , M. S. Islam , J. Phys. Chem. C 2015, 119, 15935.

[229] H. Kim , I. Park , D.‐H. Seo , S. Lee , S.‐W. Kim , W. J. Kwon , Y.‐U. Park , C. S. Kim , S. Jeon , K. Kang , J. Am. Chem. Soc. 2012, 134, 10369. 22667817 10.1021/ja3038646

[230] H. Kim , I. Park , S. Lee , H. Kim , K.‐Y. Park , Y.‐U. Park , H. Kim , J. Kim , H.‐D. Lim , W.‐S. Yoon , K. Kang , Chem. Mater. 2013, 25, 3614.

[231] H. Kim , G. Yoon , I. Park , K.‐Y. Park , B. Lee , J. Kim , Y.‐U. Park , S.‐K. Jung , H.‐D. Lim , D. Ahn , S. Lee , K. Kang , Energy Environ. Sci. 2015, 8, 3325.

[232] M. Nose , H. Nakayama , K. Nobuhara , H. Yamaguchi , S. Nakanishi , H. Iba , J. Power Sources 2013, 234, 175.

[233] H. Moriwake , A. Kuwabara , C. A. J. Fisher , M. Nose , H. Nakayama , S. Nakanishi , H. Iba , Y. Ikuhara , J. Power Sources 2016, 326, 220.

[234] M. Nose , S. Shiotani , H. Nakayama , K. Nobuhara , S. Nakanishi , H. Iba , Electrochem. Commun. 2013, 34, 266.

[235] S. Y. Lim , H. Kim , J. Chung , J. H. Lee , B. G. Kim , J.‐J. Choi , K. Y. Chung , W. Cho , S.‐J. Kim , W. A. Goddard , Y. Jung , J. W. Choi , Proc. Nat. Acad. Sci. USA 2014, 111, 599. 24379365 10.1073/pnas.1316557110 PMC3896142

[236] C. Deng , S. Zhang , ACS Appl. Mater. Interfaces 2014, 6, 9111. 24865173 10.1021/am501072j

[237] Q. Li , B. Lin , S. Zhang , C. Deng , J. Mater. Chem. A 2016, 4, 5719.

[238] H. Chen , Q. Hao , O. Zivkovic , G. Hautier , L.‐S. Du , Y. Tang , Y.‐Y. Hu , X. Ma , C. P. Grey , G. Ceder , Chem. Mater. 2013, 25, 2777.

[239] C. Wang , M. Sawicki , S. Emani , C. Liu , L. L. Shaw , Electrochim. Acta 2015, 161, 322.

[240] N. Hassanzadeh , S. K. Sadrnezhaad , G. Chen , Electrochim. Acta 2016, 208, 188.

[241] W. Huang , J. Zhou , B. Li , J. Ma , S. Tao , D. Xia , W. Chu , Z. Wu , Sci. Rep. 2014, 4, 4188. 24595232 10.1038/srep04188 PMC3942702

[242] S. I. Park , I. Gocheva , S. Okada , J.‐i. Yamaki , J. Electrochem. Soc. 2011, 158, A1067.

[243] W. Wu , A. Mohamed , J. F. Whitacre , J. Electrochem. Soc. 2013, 160, A497.

[244] G. Pang , C. Yuan , P. Nie , B. Ding , J. Zhu , X. Zhang , Nanoscale 2014, 6, 6328. 24755904 10.1039/c3nr06730k

[245] X. Li , X. Zhu , J. Liang , Z. Hou , Y. Wang , N. Lin , Y. Zhu , Y. Qian , J. Electrochem. Soc. 2014, 161, A1181.

[246] M. Vujković , M. Mitrić , S. Mentus , J. Power Sources 2015, 288, 176.

[247] J. F. Whitacre , S. Shanbhag , A. Mohamed , A. Polonsky , K. Carlisle , J. Gulakowski , W. Wu , C. Smith , L. Cooney , D. Blackwood , J. C. Dandrea , C. Truchot , Energy Technol. 2015, 3, 20.

[248] X. Wu , Y. Cao , X. Ai , J. Qian , H. Yang , Electrochem. Commun. 2013, 31, 145.

[249] Z. Li , D. Young , K. Xiang , W. C. Carter , Y.‐M. Chiang , Adv. Energy Mater. 2013, 3, 290.

[250] Z. Hou , X. Li , J. Liang , Y. Zhu , Y. Qian , J. Mater. Chem. A 2015, 3, 1400.

[251] G. Pang , P. Nie , C. Yuan , L. Shen , X. Zhang , J. Zhu , B. Ding , Energy Technol. 2014, 2, 705.

[252] W. Wu , S. Shabhag , J. Chang , A. Rutt , J. F. Whitacre , J. Electrochem. Soc. 2015, 162, A803.

[253] Q. Zhang , C. Liao , T. Zhai , H. Li , Electrochim. Acta 2016, 196, 470.

[254] K. Nakamoto , Y. Kano , A. Kitajou , S. Okada , J. Power Sources 2016, 327, 327.

[255] A. J. Fernández‐Ropero , D. Saurel , B. Acebedo , T. Rojo , M. Casas‐Cabanas , J. Power Sources 2015, 291, 40.

[256] P. R. Kumar , Y. H. Jung , B. Moorthy , D. K. Kim , J. Electrochem. Soc. 2016, 163, A1484.

[257] Y. H. Jung , C. H. Lim , J.‐H. Kim , D. K. Kim , RSC Adv. 2014, 4, 9799.

[258] L. Zhang , T. Huang , A. Yu , J. Alloys Compd. 2015, 646, 522.

[259] W. Song , X. Ji , Y. Zhu , H. Zhu , F. Li , J. Chen , F. Lu , Y. Yao , C. E. Banks , ChemElectroChem 2014, 1, 871.

[260] C. W. Mason , F. Lange , ECS Electrochem. Lett. 2015, 4, A79.

[261] C. Deng , S. Zhang , Y. Wu , Nanoscale 2015, 7, 487. 25407134 10.1039/c4nr05175k

[262] P. R. Kumar , Y. H. Jung , J. E. Wang , D. K. Kim , J. Power Sources 2016, 324, 421.

[263] S. Curtarolo , G. L. W. Hart , M. B. Nardelli , N. Mingo , S. Sanvito , O. Levy , Nat. Mater. 2013, 12, 191. 23422720 10.1038/nmat3568

[264] A. Jain , Y. Shin , K. A. Persson , Nat. Rev. Mater. 2016, 1, 15004.

[265] G. Hautier , A. Jain , S. P. Ong , B. Kang , C. Moore , R. Doe , G. Ceder , Chem. Mater. 2011, 23, 3495.

[266] A. Urban , D.‐H. Seo , G. Ceder , Npj Comput. Mater. 2016, 2, 16002.

[267] G. Hautier , A. Jain , H. Chen , C. Moore , S. P. Ong , G. Ceder , J. Mater. Chem. 2011, 21, 17147.

[268] H. Chen , G. Hautier , A. Jain , C. Moore , B. Kang , R. Doe , L. Wu , Y. Zhu , Y. Tang , G. Ceder , Chem. Mater. 2012, 24, 2009.

[269] S. P. Ong , V. L. Chevrier , G. Hautier , A. Jain , C. Moore , S. Kim , X. Ma , G. Ceder , Energy Environ. Sci. 2011, 4, 3680.

[270] M. S. Islam , C. A. J. Fisher , Chem. Soc. Rev. 2014, 43, 185. 24202440 10.1039/c3cs60199d

